# Potential impact of diabetes prevention on mortality and future burden of dementia and disability: a modelling study

**DOI:** 10.1007/s00125-019-05015-4

**Published:** 2019-11-15

**Authors:** Piotr Bandosz, Sara Ahmadi-Abhari, Maria Guzman-Castillo, Jonathan Pearson-Stuttard, Brendan Collins, Hannah Whittaker, Martin J. Shipley, Simon Capewell, Eric J. Brunner, Martin O’Flaherty

**Affiliations:** 1 Department of Public Health and Policy, University of Liverpool, 3rd Floor, Whelan Building, Brownlow Hill, Liverpool, L69 3GB UK; 2 Department of Prevention and Medical Education, Medical University of Gdansk, Gdansk, Poland; 3 Institute of Epidemiology and Health Care, University College London, London, UK; 4 Ageing Epidemiology (AGE) Research Unit, Imperial College London, London, UK; 5 Department of Social Sciences, University of Helsinki, Helsinki, Finland; 6 School of Public Health, Imperial College London, London, UK; 7 National Heart and Lung Institute (NHLI), Imperial College London, London, UK

**Keywords:** Dementia, Diabetes, Disability, Forecast, Modelling study

## Abstract

**Aims/hypothesis:**

Diabetes is associated with an increased risk of dementia. We estimated the potential impact of trends in diabetes prevalence upon mortality and the future burden of dementia and disability in England and Wales.

**Methods:**

We used a probabilistic multi-state, open cohort Markov model to integrate observed trends in diabetes, cardiovascular disease and dementia to forecast the occurrence of disability and dementia up to the year 2060. Model input data were taken from the English Longitudinal Study of Ageing, Office for National Statistics vital data and published effect estimates for health-state transition probabilities. The baseline scenario corresponded to recent trends in obesity: a 26% increase in the number of people with diabetes by 2060. This scenario was evaluated against three alternative projected trends in diabetes: increases of 49%, 20% and 7%.

**Results:**

Our results suggest that changes in the trend in diabetes prevalence will lead to changes in mortality and incidence of dementia and disability, which will become visible after 10–15 years. If the relative prevalence of diabetes increases 49% by 2060, expected additional deaths would be approximately 255,000 (95% uncertainty interval [UI] 236,000–272,200), with 85,900 (71,500–101,600) cumulative additional cases of dementia and 104,900 (85,900–125,400) additional cases of disability. With a smaller relative increase in diabetes prevalence (7% increase by 2060), we estimated 222,200 (205,700–237,300) fewer deaths, and 77,000 (64,300–90,800) and 93,300 (76,700–111,400) fewer additional cases of dementia and disability, respectively, than the baseline case of a 26% increase in diabetes.

**Conclusions/interpretation:**

Reducing the burden of diabetes could result in substantial reductions in the incidence of dementia and disability over the medium to long term.

**Electronic supplementary material:**

The online version of this article (10.1007/s00125-019-05015-4) contains peer-reviewed but unedited supplementary supplementary material, which is available to authorised users.

## Introduction

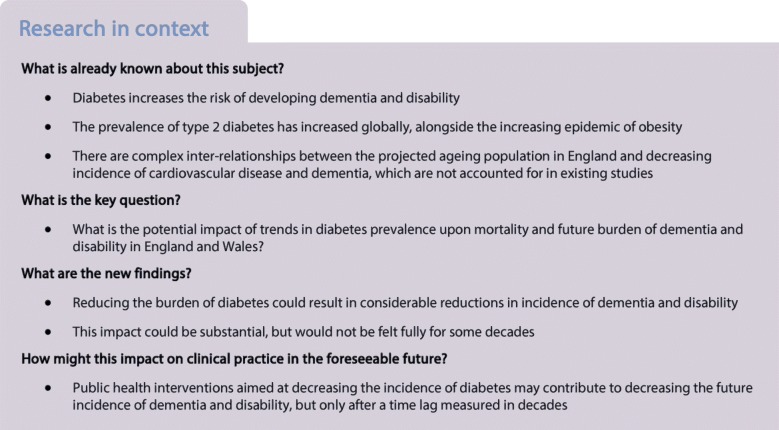



The rapid ageing of populations in high-income countries has triggered major concerns regarding the future burden of age-related chronic diseases. In England and Wales, there are around 800,000 people living with dementia, and this number is set to increase by 60% by 2040 [1]. A substantial increase is also predicted for the number of people living with any functional impairment (disability). This constitutes a major policy challenge [2].

Currently, there is no dementia treatment that can significantly modify disease progression. However, several modifiable risk factors for dementia and disability have been identified, thus creating the possibility for prevention. Up to 35% of cases of dementia are attributable to a combination of nine risk factors: low educational attainment, midlife hypertension, midlife obesity, hearing loss, late-life depression, diabetes, physical inactivity, smoking and social isolation [3].

Reducing these risk factors can have a substantial impact on the risk of developing dementia. During 25 years of follow-up in a large cohort study in the USA, dementia incidence was approximately 80% higher in those with diabetes compared with those without diabetes [4]. Among individuals in late midlife, those with diabetes had a 24% faster cognitive decline than those without diabetes [5]. For disability, the Global Burden of Disease Study showed that diabetes was the sixth most common cause of disability in 2015 [2]. England and Wales have recently experienced favourable declines in smoking prevalence and BP levels. These possibly explain the 2.7% annual decrease in dementia incidence observed in the last two decades [1]. However, there is evidence of worsening trends in other, potentially important, risk factors for both dementia and disability. The prevalence of adult obesity increased from 15% in 1993 to 26% in 2010 before recently plateauing [6]. Type 2 diabetes prevalence, driven mainly by obesity trends, more than doubled between 1993 and 2015 and is expected to increase further from 8.6% in 2015 to 9.7% by 2035, generating more than 1.1 million additional cases in England [7, 8]. Midlife obesity and type 2 diabetes have both been shown to be related to dementia and disability incidence, based on evidence from observational studies [2, 9, 10].

The UK government has responded with prevention efforts to reduce the burden of obesity and diabetes. These include the Diabetes Prevention Programme which targets individuals at highest risk. There have also been population-based policy approaches to prevent obesity such as the soft drinks industry levy, sugar and ‘calorie’ reduction programmes, and national marketing campaigns [11, 12]. Despite these efforts, future trends in obesity and diabetes prevalence remain uncertain. Correspondingly, the burdens of dementia and disability, including mortality rates, are unknown.

In this study, we aimed to estimate the potential effects of future trends in diabetes prevalence on mortality outcomes and the future burden of dementia and disability in England and Wales up to 2060.

## Methods

We conducted our analyses in two stages: first, we estimated plausible future trends in diabetes prevalence given different futures for the obesity trends using the Diabetes Prevalence Model (DPM) developed by Public Health England (PHE).

**Fig. 1:**
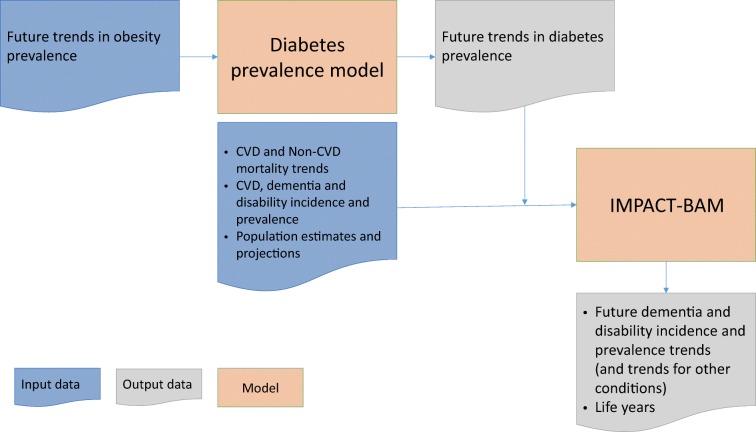
Diagram illustrating steps of the analysis

Second, we estimated future trends in diabetes prevalence and examined their potential effects upon dementia and disability over the period 2015–2060 in the England and Wales population using our previously validated IMPACT Better Ageing Model (IMPACT-BAM) [1, 13]. The flowchart in Fig. 1 describes how these two steps are connected.

### Future diabetes trends

In the first step, we examined four plausible future diabetes prevalence scenarios based on obesity prevalence projections. We assumed different futures for the currently increasing obesity trends—no change, a further acceleration, a halt, and finally, a reverse—and translated these into expected future trends in diabetes prevalence using the DPM [7]. We used this model to estimate possible future trends in diabetes prevalence given the four different futures for the currently increasing obesity trends, ranging from a 5 yearly change in the obesity prevalence of −3 to +5%. Each one of the projected trends in diabetes prevalence from 2015–2060 was used as a different scenario to be explored later in IMPACT-BAM (see electronic supplementary material [ESM] Chapter 4.4 and ESM Fig. 12).

The baseline scenario assumed a continuation of recent trends in leading diabetes drivers, mainly an increase in obesity prevalence of 1% every 5 years. This obesity trend corresponds roughly to the last 5 years of change in obesity reported in the Health Survey for England study [8]. For this trend, the DPM predicted a 26% rise in diabetes prevalence from 8.6% in 2015 to 10.8% in 2060.

Three additional scenarios, based on current trends in drivers of diabetes (a further acceleration, a halt, and a reverse in the current obesity trend) were evaluated against the baseline scenario. These scenarios were: (A) an increase in relative diabetes prevalence of 49%; (B) an increase by 20%; and (C) a slowing down of the increase to 7% by 2060. Details of all modelled scenarios are presented in Table 1 and ESM Chapter 4.4.

**Table 1:** Predicted prevalence of type 2 diabetes by calendar year for four scenarios of trends in obesity, compared with the baseline scenario

Scenario	Change in obesity prevalence (%/5 years)	Change in diabetes prevalence by 2060 (%)	Calendar year					
			2015	2020	2030	2040	2050	2060
			Diabetes prevalence (%)					
A	+5	+49	8.6	9.1	10.1	11.1	12.0	12.8
Baseline^a^	+1	+26	8.6	9.0	9.6	10.1	10.5	10.8
B	0	+20	8.6	8.9	9.5	9.9	10.2	10.3
C	−3	+7	8.6	8.8	9.1	9.3	9.3	9.2

Projection of diabetes prevalence based on estimates from PHE DPM using different assumptions of future obesity trends [7]

^a^Baseline scenario is that the current trend in the prevalence of obesity will continue

### Effect of diabetes trends in dementia and disability

#### Overview of the IMPACT-BAM model

We extended the IMPACT-BAM to estimate the potential effects of the plausible changing trends in diabetes prevalence upon dementia and disability over the period 2015–2060 in the England and Wales population.

IMPACT-BAM is a probabilistic multi-state, open cohort Markov model which follows the progression of a healthy population (aged ≥35 years) of England and Wales from 2006 to 2060 into eight different health states characterised by the presence or absence of cardiovascular disease (CVD), cognitive impairment, dementia and moderate to severe disability, and two states for death from CVD and non-CVD causes (ESM Fig. 1).

Before running the simulation, we populated each state in the model based on the Office for National Statistics population estimates in 2006 (start year) and prevalence of the above conditions from the English Longitudinal Study of Ageing (ELSA), except for the new cohort of 35-year-olds that enters the system through the disease-free state. The simulation allows individuals to move to other states in the model. The arrows in ESM Fig. 1 indicate the possible movements of people between these ten states, which are governed by one-year probabilities of transition. For example, a healthy 55-year-old man starts the simulation in state 1 (disease-free state) in 2006. He moves to state 2 (CVD) in 2007 after having a stroke. In 2008 he could either die from complications of the stroke (he moves to state 9), die from any other causes (he moves to state 10), or he could develop cognitive impairment (moving to state 3) or disability (moving to state 5). As above, movements to any state are driven by transition probabilities.

Transition probabilities were previously calculated using combined data from the ELSA and mortality projections. These projections were estimated separately for cardiovascular and non-cardiovascular mortality rates based on observed data reported by the Office for National Statistics up to 2016. P-spline smoothed lines [14–16] were fitted to logarithmic transformed CVD and non-CVD mortality rates in each 5-year age band from 1990 to 2016 by sex using the p-spline function in Stata software. Additional details are provided in the ESM Chapter 2 and ESM Figs 2–5.

We defined disability as the inability to independently carry out one or more activities of daily living, which included getting in or out of bed, walking across a room, bathing or showering, using the toilet, dressing, cutting food and eating. This definition of disability captures individuals who have difficulty maintaining independence and require supportive care.

Dementia was defined on the basis of the coexistence of cognitive impairment and disability, or a report of a doctor diagnosis of dementia by the participant or caregiver. Cognitive impairment was defined as an impairment in two or more functional tests (such as orientation to time, immediate and delayed memory, verbal fluency and numeracy function) or a score higher than 3.6 on the Informant Questionnaire on Cognitive Decline (IQCODE) [17].

A more detailed overview of the model can be found in the previously published papers [1, 13] and ESM Chapters 1–2 and ESM Figs 1–5. Information about model validation is provided in ESM Chapter 3 and Figs 6–9. A general description of these types of Markov models can be found in the paper by Briggs and Sculpher [18].

#### Diabetes trends effect on transition probabilities

For this study, we assumed that trends in the prevalence of type 2 diabetes from each scenario, calculated in the first stage, would modify some of the IMPACT-BAM transition probabilities, which ultimately would result in changes in the burden of dementia and disability. We assumed that the affected transition probabilities were those representing the risk of CVD and non-CVD death, cognitive decline incidence, CVD incidence, disability incidence and recovery from disability. To model these changes in transition probabilities, we used an approach based on the population attributable risk fraction (PARF). The PARF calculates the proportion by which disease burden would be reduced if there were no diabetes in the population. A simpler version of this approach has been used previously in other IMPACT models [19, 20].

Previous research has demonstrated that the association between diabetes and cognitive decline and CHD is dependent upon diabetes duration [21]. This is consistent with the view that the presence of vascular risk factors and insulin resistance in midlife increase the risk not only of developing diabetes and atherosclerosis but also of cognitive decline and dementia later in life. The development of hyperglycaemia, glucose intolerance, microvascular and macrovascular complications that are associated with type 2 diabetes then further accelerate diabetes-associated cognitive decline.

To account for the effect of diabetes duration, we used the extended formula of PARF to account for multi-category exposures [22]. The different categories of exposure represent six different categories of lengths of time living with the disease: diabetes duration of fewer than 5 years; 5–9 years; 10–14 years; 15–19 years; 20–24 years and more than 25 years.

The extended PARF formula requires: (1) age- and sex-specific estimates of the risk factor prevalence at each exposure level; and (2) RRs comparing every exposure level with the unexposed group (i.e. stratified by age, sex and affected transition probability). We used the 2014 Health Survey for England (HSE) data to obtain age- and sex-specific distributions of diabetes prevalence across the six categories of diabetes duration. For the baseline scenario, we assumed that the prevalence in each of the categories would remain constant in the future. For the rest of the scenarios, we modified the prevalence in each category across time to match the ageing of the population (more details are provided in ESM Chapter 4.5 and Figs 13–20) We obtained the RRs from published studies and by carrying out meta-analyses (see ESM Chapter 4, ESM Figs 10–11 and ESM Table 1). All RRs used had been adjusted for BMI. Then, we adjusted these RRs by diabetes duration using the results from the ADVANCE trial, which quantifies the risk for macro- and microvascular complications and all-cause death for each 5 year increase in diabetes duration [23].

Ultimately, we were interested in how the PARF changes because of trends in diabetes prevalence. Symbolically, this would be:

$$ \Delta \mathrm{PARF}=\mathrm{PARF}-\mathrm{PARF}^{\prime } $$

where PARF′ is the calculated PARF for the (lower) diabetes prevalence from the baseline scenario.

#### Outcomes

For each scenario, the ΔPARF was then multiplied by the appropriate transition probability to generate a new transition probability. We repeated this procedure for all the transition probabilities. We then recalculated the IMPACT model with the new set of transition probabilities and generated scenario-specific numbers of cases of dementia, disability and deaths from 2016 onwards and compared them against the baseline scenario (i.e. the model with the original transition probability values).

Finally, we reported the cumulative number of new cases of dementia, disability and deaths attributable to each scenario for the population aged 65 and over, in comparison with the baseline scenario. We also presented life years gained (LYG) for each scenario and the proportion of life spent with disability.

#### Sensitivity analysis

To explore the impact of parameter uncertainty on model outputs, we conducted a probabilistic sensitivity analysis using Monte Carlo simulation. The procedure entailed iterative sampling from specified distributions for the input parameters that were used in the model, and then re-calculation of the outputs. We performed 1000 iterations to estimate 95% uncertainty intervals (95% UIs) for the output variables.

The model was developed as a package in R software (version 3.4.2, https://cran.r-project.org). Detailed information on preparing scenarios data and methods used to evaluated the effect of diabetes on model transition probabilities can be found in ESM Chapters 4–6 and ESM Figs 10–20.

## Results

### Baseline scenario

Our IMPACT-BAM baseline results (see Table 2) suggested that the number of disability incident cases among the population aged 65 and over is expected to increase from approximately 223,600 (95% UI 219,200–228,100) per year in 2015 to 288,100 (275,900–300,200) in 2045 and then fall slightly to 271,600 (253,900–287,400) in 2060. The number of incident dementia cases is also expected to rise from approximately 142,000 (137,000–147,300) per year in 2015 to 208,500 (197,400–218,900) in 2045 and 203,700 (189,100–217,500) in 2060.

**Table 2:** Projected number and rate of incident cases of disability, dementia and mortality in the England and Wales population aged ≥65 in 2030, 2045 and 2060, compared with those observed in 2015 for the baseline scenario

Sex	Year	Disability incident cases		Dementia incident cases		Total deaths	
		Number (thousands)	Per 1000 population	Number (thousands)	Per 1000 population	Number (thousands)	Per 1000 population
All	2015	224 (219 to 228)	21.4 (21.0 to 21.9)	142 (137 to 147)	13.6 (13.1 to 14.1)	404 (401 to 408)	38.7 (38.3 to 39.2)
	2030	269 (261 to 276)	19.3 (18.8 to 19.8)	187 (179 to 194)	13.4 (12.9 to 13.9)	442 (436 to 449)	31.7 (30.9 to 32.6)
	2045	288 (276 to 300)	17.1 (16.6 to 17.6)	209 (197 to 219)	12.4 (11.8 to 12.9)	471 (466 to 478)	27.9 (27.0 to 29.1)
	2060	272 (254 to 287)	13.9 (13.3 to 14.4)	204 (189 to 218)	10.4 (9.9 to 10.9)	462 (458 to 468)	23.6 (22.7 to 25.0)
Men	2015	100 (96.1 to 104)	21.2 (20.3 to 22.1)	61 (57 to 66)	13.0 (12.0 to 14.0)	192 (190 to 194)	40.6 (40.0 to 41.1)
	2030	126 (120 to 132)	19.4 (18.4 to 20.3)	86 (80 to 92)	13.2 (12.2 to 14.3)	217 (214 to 220)	33.3 (32.5 to 34.3)
	2045	137 (128 to 146)	17.1 (16.1 to 18.1)	99 (91 to 107)	12.3 (11.3 to 13.3)	233 (230 to 236)	29.0 (27.9 to 30.2)
	2060	132 (120 to 142)	13.8 (12.8 to 14.8)	99 (88 to 109)	10.4 (9.4 to 11.4)	232 (230 to 235)	24.3 (23.4 to 25.7)
Women	2015	123 (121 to 126)	21.6 (21.2 to 22.0)	81 (79 to 83)	14.1 (13.8 to 14.5)	213 (211 to 215)	37.2 (36.8 to 37.7)
	2030	143 (139 to 147)	19.2 (18.8 to 19.7)	101 (97 to 104)	13.6 (13.1 to 14.0)	225 (222 to 229)	30.3 (29.5 to 31.2)
	2045	151 (145 to 156)	17.1 (16.6 to 17.5)	110 (105 to 114)	12.4 (11.9 to 12.9)	238 (236 to 242)	26.9 (26.1 to 28.1)
	2060	140 (132 to 147)	14.0 (13.6 to 14.3)	105 (99 to 110)	10.5 (10.1 to 10.9)	230 (228 to 233)	22.9 (22.1 to 24.3)

The baseline scenario is that the current trend in the prevalence of diabetes will continue, which will result in a 26% increase in diabetes prevalence by 2060

95% UIs are shown in brackets

This increase in the number of annual incident cases occurred despite a continuous decline in the incidence rates of dementia and disability. This suggests that the reason for the increasing number of projected new cases of dementia and disability is the projected ageing of the population, due to increasing longevity. The incidence of disability expressed per 1000 population aged ≥65 is forecasted to decrease from approximately 21.4 (95% UI 21.0–21.9) per 1000 person-years in 2015 to 17.1 (16.6–17.6) in 2045 and then 13.9 (13.3–14.4) in 2060. This decrease is also observed for dementia, with an expected fall from 13.6 (13.1–14.1) per 1000 person-years in 2015 to 12.4 (11.8–12.9) in 2045 and 10.4 (9.9–10.9) in 2060.

The projected total mortality rate in people aged ≥65 is predicted to decrease by approximately 39% between 2015 and 2060, from 38.7 (95% UI 38.3–39.2) to 23.6 (22.7–25.0) per 1000 population.

### Comparison of three future scenarios

#### Effect of diabetes trends on projected mortality

For the most adverse scenario (A) we might expect about 79,700 (95% UI 72,500–86,000) additional deaths attributable to diabetes by 2045 (43.4 deaths per 100,000 population) and approximately 255,000 (236,000–272,200) additional deaths (79.5 per 100,000) by 2060 (Table 3). This corresponds to approximately 650,000 (580,000–700,000) cumulative life years lost by 2045 and 2,570,000 (2,350,000–2,770,000) by 2060, which is 0.9% of life years lost for 2060 in comparison with the baseline scenario.

**Table 3:** Number of deaths avoided for scenarios A, B and C vs baseline scenario: England and Wales, population aged ≥65

Sex	Calendar year	Scenario A (49% increase in diabetes prevalence by 2060)		Scenario B (20% increase in diabetes prevalence by 2060)		Scenario C (7% increase in diabetes prevalence by 2060)	
		Deaths avoided (thousands)^a^ (cumulative since 2015)	Per 100,000 population^a^	Deaths avoided (thousands) (cumulative since 2015)	Per 100,000 population	Deaths avoided (thousands) (cumulative since 2015)	Per 100,000 population
All	2030	−12.4 (−13.7 to −10.9)	−14.1 (−15.5 to −12.6)	3.1 (2.7 to 3.4)	3.4 (3.0 to 3.7)	12.0 (10.6 to 13.3)	13.3 (11.9 to 14.6)
	2045	−79.7 (−86.0 to −72.5)	−43.4 (−46.6 to −40.0)	18.9 (17.2 to 20.4)	10.1 (9.3 to 10.8)	73.0 (66.5 to 78.8)	38.5 (35.5 to 41.3)
	2060	−255.0 (−272.2 to −236.0)	−79.5 (−84.9 to −73.8)	58.6 (54.3 to 62.6)	17.7 (16.5 to 18.9)	222.2 (205.7 to 237.3)	65.5 (60.8 to 70.0)
Men	2030	−6.7 (−7.4 to −5.9)	−16.3 (−17.8 to −14.5)	1.6 (1.5 to 1.8)	3.9 (3.5 to 4.3)	6.5 (5.7 to 7.2)	15.4 (13.8 to 16.8)
	2045	−42.5 (−45.8 to −38.7)	−48.7 (−52.2 to −44.8)	10.1 (9.2 to 10.9)	11.3 (10.4 to 12.1)	39.0 (35.5 to 42.0)	43.2 (39.8 to 46.2)
	2060	−135.8 (−144.8 to −126.1)	−86.9 (−92.7 to −80.9)	31.2 (29.0 to 33.3)	19.4 (18.1 to 20.7)	118.4 (110.0 to 126.3)	71.4 (66.5 to 76.3)
Women	2030	−5.7 (−6.3 to −5.0)	−12.2 (−13.4 to −10.9)	1.4 (1.2 to 1.5)	2.9 (2.6 to 3.2)	5.5 (4.9 to 6.1)	11.6 (10.3 to 12.7)
	2045	−37.2 (−40.2 to −33.7)	−38.7 (−41.6 to −35.5)	8.8 (8.0 to 9.5)	9.0 (8.3 to 9.7)	34.0 (30.9 to 36.8)	34.3 (31.5 to 36.9)
	2060	−119.2 (−127.5 to −109.9)	−72.3 (−77.5 to −67.0)	27.4 (25.2 to 29.3)	16.1 (14.9 to 17.3)	103.8 (95.8 to 111.0)	59.8 (55.4 to 64.1)

The baseline scenario is that the current trend in the prevalence of diabetes will continue, which will result in a 26% increase in diabetes prevalence by 2060

95% UIs are shown in brackets

^a^Negative values indicate additional burden

In contrast, for scenario B we might expect about 18,900 (95% UI 17,200–20,400) fewer deaths due to diabetes by 2045 and 58,600 (54,300–62,600) by 2060 in comparison with the baseline scenario. This resulted in projected LYG of 150,000 (140,000–170,000) and 600,000 (550,000–650,000) by 2045 and 2060, respectively. This corresponds to a 0.2% increase in the number of life years in comparison with the baseline scenario in 2060.

**Fig. 2:**
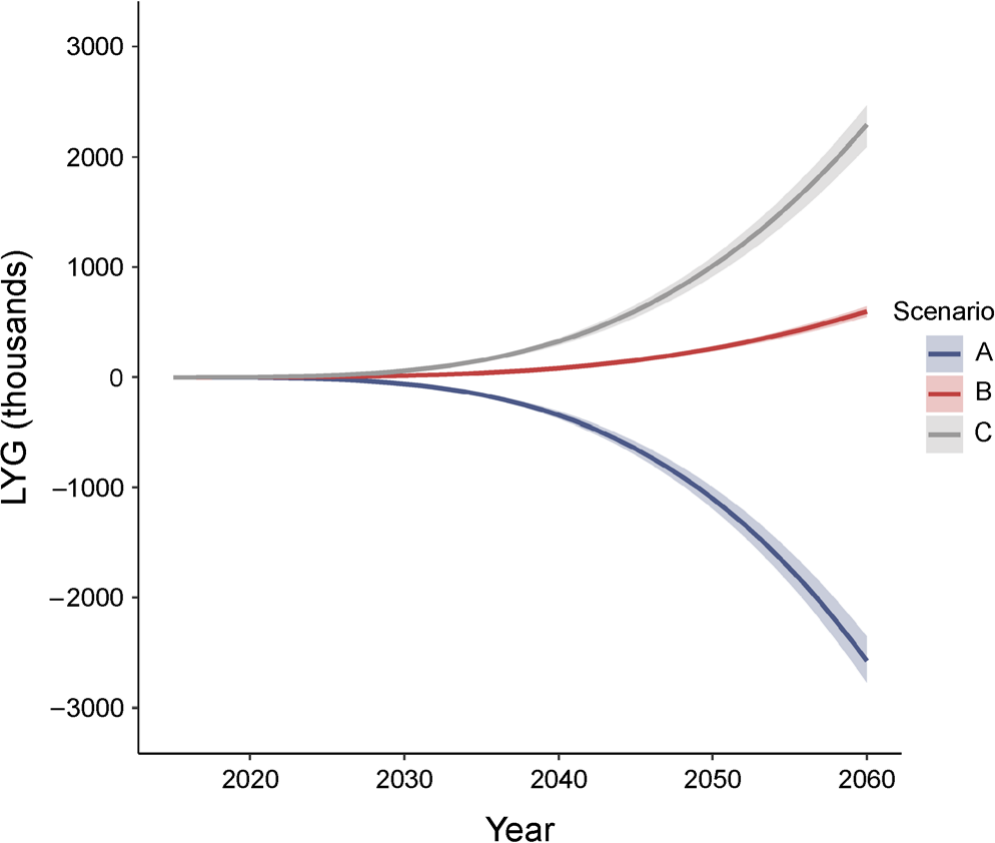
Cumulative number of LYG for modelled scenarios (thousands) for England and Wales, population aged ≥65. The shading represents 95% UIs. Scenarios: A, 49% increase in diabetes by 2060; B, 20% increase in diabetes by 2060; C, 7% increase in diabetes by 2060

For the most optimistic scenario (C), we might expect approximately 73,000 (95% UI 66,500–78,800) and 222,200 (205,700–237,300) fewer deaths in comparison with the baseline scenario, for 2045 and 2060, respectively (Table 3). This results in gaining approximately 600,000 (540,000–660,000) additional life years by 2045 and 2,290,000 (2,090,000–2,470,000) life years by 2060 (Fig. 2), an increase in life years of 0.8% in comparison with the baseline scenario in 2060.

#### Effect of diabetes trends on disability and dementia

Our results suggest that any changes in diabetes prevalence will have almost no effect on dementia and disability burden in the next 5 years (see Table 4). However, these effects increase significantly during subsequent decades. For scenario A (49% increase in diabetes up to 2060), we might expect approximately 104,900 (95% UI 85,900–125,400) additional cases of disability and some 85,900 (71,500–101,600) additional cases of dementia before 2060. On the other hand, if there is a deceleration in the rate of increase of diabetes prevalence (scenario B), we might avoid approximately 24,400 (20,000–29,200) new cases of disability and 20,100 (16,800–23,700) new cases of dementia cumulatively by 2060.

**Table 4:** Number of new cases of disability and dementia avoided for scenarios A, B and C vs baseline scenario: England and Wales, population aged ≥65

Sex	Calendar year	Scenario A (49% increase in diabetes prevalence by 2060)		Scenario B (20% increase in diabetes prevalence by 2060)		Scenario C (7% increase in diabetes prevalence by 2060)	
		Cases avoided (thousands)^a^ (cumulative since 2015)	Per 100,000 population	Cases avoided (thousands)(cumulative since 2015)	Per 100,000 population	Cases avoided (thousands)(cumulative since 2015)	Per 100,000 population
Disability							
All	2030	−5.0 (−6.2 to −3.8)	−5.9 (−7.2 to −4.5)	1.2 (0.8 to 1.5)	1.4 (1.1 to 1.7)	4.7 (3.6 to 5.9)	5.6 (4.3 to 6.8)
	2045	−33.8 (−40.6 to −27.1)	−18.6 (−21.9 to −15.4)	8.0 (6.4 to 9.6)	4.4 (3.6 to 5.1)	31.1 (25.1 to 37.4)	16.8 (14.0 to 19.6)
	2060	−104.9 (−125.4 to −85.9)	−30.9 (−36.3 to −25.9)	24.4 (20.0 to 29.2)	7.1 (5.9 to 8.3)	93.3 (76.7 to 111.4)	26.4 (22.2 to 31.1)
Men	2030	−2.6 (−3.3 to −2.0)	−6.6 (−8.0 to −5.1)	0.6 (0.3 to 0.7)	1.6 (1.2 to 1.9)	2.4 (1.8 to 3.0)	6.2 (4.8 to 7.6)
	2045	−17.8 (−21.4 to −14.3)	−20.8 (−24.6 to −17.3)	4.2 (3.3 to 5.0)	4.9 (4.1 to 5.8)	16.3 (13.2 to 19.6)	18.8 (15.7 to 22.1)
	2060	−56.2 (−67.0 to −46.0)	−34.6 (−40.7 to −29.2)	13.0 (10.7 to 15.5)	7.9 (6.7 to 9.3)	50.0 (41.0 to 59.7)	29.5 (24.9 to 34.7)
Women	2030	−2.3 (−2.9 to −1.8)	−5.3 (−6.4 to −4.1)	0.6 (0.4 to 0.7)	1.3 (1.0 to 1.6)	2.3 (1.7 to 2.8)	5.0 (3.9 to 6.1)
	2045	−16.0 (−19.3 to −12.8)	−16.6 (−19.6 to −13.7)	3.8 (3.1 to 4.6)	3.9 (3.2 to 4.6)	14.7 (11.9 to 17.7)	15.0 (12.4 to 17.6)
	2060	−48.9 (−58.4 to −40.0)	−27.5 (−32.3 to −22.9)	11.4 (9.3 to 13.6)	6.3 (5.2 to 7.4)	43.5 (35.7 to 52.0)	23.7 (19.7 to 27.7)
Dementia							
All	2030	−3.4 (−4.3 to −2.6)	−4.2 (−5.1 to −3.3)	0.8 (0.5 to 1.0)	1.0 (0.8 to 1.2)	3.2 (2.4 to 4.0)	4.0 (3.1 to 4.9)
	2045	−25.7 (−30.7 to −20.9)	−15.1 (−17.6 to −12.6)	6.1 (4.9 to 7.3)	3.6 (3.0 to 4.1)	23.8 (19.4 to 28.4)	13.7 (11.5 to 16.0)
	2060	−85.9 (−101.6 to −71.5)	−26.7 (−31.0 to −22.8)	20.1 (16.8 to 23.7)	6.2 (5.3 to 7.1)	77.0 (64.3 to 90.8)	23.1 (19.7 to 26.8)
Men	2030	−1.8 (−2.3 to −1.3)	−4.6 (−5.6 to −3.6)	0.4 (0.1 to 0.5)	1.1 (0.9 to 1.4)	1.6 (1.2 to 2.0)	4.4 (3.4 to 5.4)
	2045	−13.5 (−16.2 to −11.0)	−17.0 (−19.8 to −14.1)	3.2 (2.5 to 3.8)	4.0 (3.3 to 4.7)	12.5 (10.1 to 15.0)	15.5 (12.9 to 18.0)
	2060	−46.5 (−54.9 to −38.5)	−30.2 (−35.2 to −25.6)	10.8 (9.0 to 12.8)	7.0 (5.9 to 8.1)	41.6 (34.6 to 49.1)	26.0 (22.0 to 30.3)
Women	2030	−1.7 (−2.1 to −1.2)	−3.8 (−4.7 to −3.0)	0.4 (0.3 to 0.5)	0.9 (0.7 to 1.1)	1.6 (1.2 to 2.0)	3.6 (2.8 to 4.5)
	2045	−12.2 (−14.6 to −9.9)	−13.4 (−15.7 to −11.2)	2.9 (2.4 to 3.5)	3.2 (2.6 to 3.7)	11.4 (9.2 to 13.5)	12.2 (10.2 to 14.2)
	2060	−39.8 (−47.1 to −32.8)	−23.5 (−27.4 to −19.8)	9.3 (7.7 to 11.0)	5.4 (4.6 to 6.3)	35.6 (29.5 to 42.2)	20.4 (17.2 to 23.6)

The baseline scenario is that the current trend in diabetes will continue, which will result in a 26% increase in diabetes prevalence by 2060

95% UIs are shown in brackets

^a^Negative values for number of cases avoided mean extra additional cases

The most optimistic scenario (C) might result in approximately 93,300 (76,700–111,400) fewer cumulative new cases of disability and 77,000 (64,300–90,800) fewer cases of dementia by 2060. This corresponds to 2.2% fewer new cases of dementia and 1.9% fewer cases of disability in comparison with the baseline scenario in 2060.

#### Compression of morbidity

**Fig. 3:**
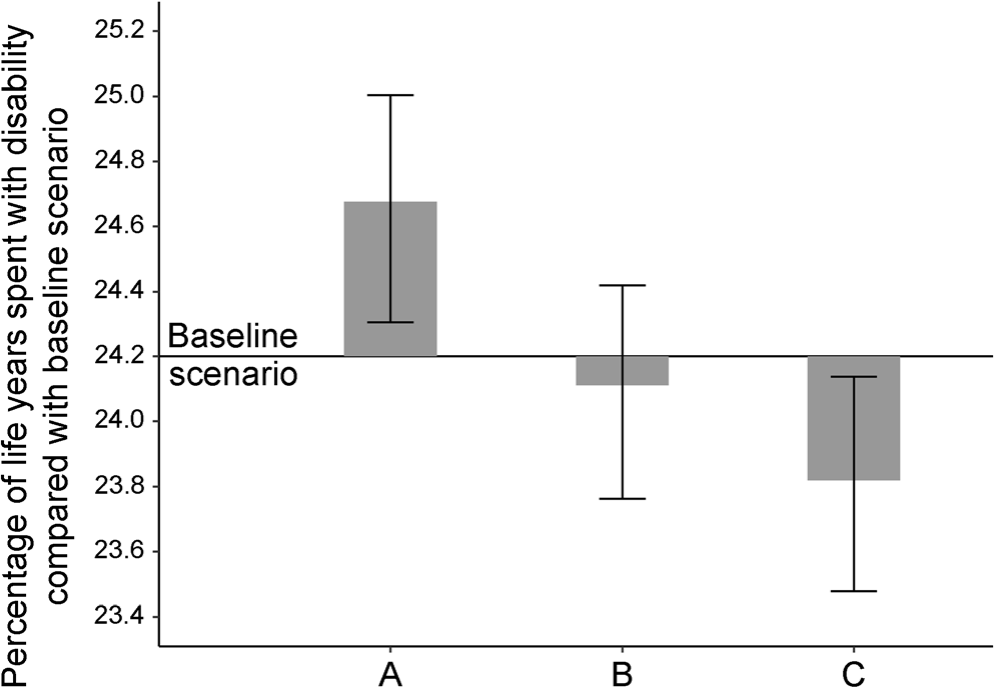
Projected percentage of life years spent with disability in 2060 for England and Wales, population aged ≥65. In the baseline scenario, 24.2% of life years were spent with disability (horizontal solid line). Bars represent corresponding proportions for scenarios A, B and C. Error bars represent 95% UIs. Scenarios: A, 49% increase in diabetes by 2060; B, 20% increase in diabetes by 2060; C, 7% increase in diabetes by 2060

The projected trends in diabetes prevalence appeared to affect the proportion of life years spent with disability. The percentage of life years in the population spent with disability was 0.4% lower for scenario C (most optimistic) than the baseline scenario, and 0.9% lower than scenario A (most pessimistic) (Fig. 3).

## Discussion

### Key findings

Our study suggests that a reduction in diabetes prevalence would result in a decline in age-specific incidence of dementia and disability and postpone these conditions to later years in life. However, trends in the incidence of dementia and disability at the population level would not be observed for a decade as a result of decreases in mortality and prolongation of life. Crucially, our study also suggests that substantial reductions in diabetes prevalence would compress morbidity, decreasing the percentage of life spent with disability by approximately 0.4% (see Fig. 3).

Our study is the first, to our knowledge, to estimate the potential effect of future diabetes trends on the future UK burden of disability and dementia while taking into account the complex population dynamics of morbidity and mortality.

Barnes and Yaffe [24] estimated the fraction of cases of Alzheimer’s disease attributable to diabetes by calculating its population attributable risk (PAR) as 2% worldwide and 3% in the USA. This corresponds to 81,000 and 17,000 fewer cases of dementia each year worldwide and in the USA, respectively, if diabetes prevalence decreases by 10%. Our results are far more conservative. It is likely that Barnes and Yaffe’s analysis over-estimated the potential burdens, because it did not account for any observed trends in risk factors, mortality or dementia, nor consider competing risks of mortality from CVD as opposed to dementia. Moreover, these analyses, based on simple PAR approaches, did not account for any relationship between diabetes duration and outcomes. Several studies have reported that the RR of fatal CHD increases in individuals with diabetes as the years lived with the disease increases [21, 23, 25].

### Strengths and limitations

The IMPACT-BAM model accounts for complex epidemiological interactions between several important sources of morbidity like CVD, dementia and disability, which share some risk factors. The structure of the model allowed us to take into account the risks between these conditions and competing risks of dying from cardiovascular and non-cardiovascular causes. Diabetes increases the risk of dying, not only from CVD, but also from many other diseases, including several cancers. Given the declining population risk of CVD mortality in future years, the competing risk of non-CVDs is expected to become critical. Our model accounts for this by separating projections of future cardiovascular and non-cardiovascular risk of death, which represents a substantial improvement on previous methodologies [24, 26]. Specifically, we account for trends in CVD- and non-CVD mortality and incidence, trends in dementia incidence, competing risks between cardiovascular and non-cardiovascular risk factors. Our methods also account for the increase in risk of both CVD and dementia caused by the time spent living with diabetes. IMPACT-BAM also models the time lag between newly developed diabetes and its complications. To our knowledge, there are no previous studies modelling the effect of prevention of diabetes on dementia and disability which also take into consideration the time needed to develop diabetes complications. This makes our estimates closer to real-life settings. Finally, our prediction model estimates the positive and negative effects of changes in diabetes prevalence. Our scenarios of possible future diabetes burden were based on reasonable assumptions of future obesity trends.

Our study also has limitations. Our approach does not account for any effect of change in the obesity distribution in the population not mediated through diabetes, including the effect on hypertension prevalence. This means that our results may be conservative. However, obesity is a less strong risk factor for dementia than diabetes [4].

We used the pre-existing model published by PHE, the DPM, to calculate expected trends in diabetes prevalence if the obesity prevalence increase accelerates (scenario A), stops (scenario B) or reverses (scenario C). Although obesity is widely recognised as the main driver of the current diabetes epidemic, diabetes trends can also be driven by many other risk factors that may have contrasting trends such as physical activity or pharmacological intervention in individuals with impaired glucose tolerance [27]. In addition, the DPM model (available on PHE official website) lacks information on any potential methodological limitations. Moreover, our approach to projecting the future distribution of the diabetes duration in the population does not account for increased risk of death due to longer duration of diabetes. This can lead to overestimation of the number of participants with longer duration of diabetes, and overestimating the total effect of a change in diabetes prevalence on dementia and disability incidence.

We used a multi-exposure, PARF approach to translate trends in diabetes prevalence to changes in the risk of dementia and cognitive impairment. This approach assumes causality of risk factor and disease. The evidence is still not as strong as it is for the association between some other risk factors and diseases. However, there is increasing evidence of the association between diabetes and dementia [4, 28–31].

Finally, all the disability caused by conditions other than dementia and CVD is aggregated into a single model state. As a consequence, the transition probabilities of moving from this state to other states are not specific for these conditions, but rather represent an average probability for all other causes of disability. Nevertheless, because the ELSA sample is representative for the population of England and Wales, the estimated combined probabilities should be generalisable.

### Public health implications

Our findings have implications for the medium and long term. With a 35-year perspective we might expect a shift in the burden of diabetes consequences, from CVD towards a much broader range of sequelae including dementia. Our study suggests that preventing diabetes is important, not only for future CVD, but also for the dementia and disability burden. Moreover, as CVD mortality continues to decline, we expect a further shift in the burden of diabetes to non-cardiovascular complications. Contrary to some other diabetes complications, such as nephropathy or retinopathy, people with cognitive decline and dementia cannot be offered treatment to significantly slow down the progress of the disease. Since an older population structure is the leading reason for the increase in the dementia burden, it is reasonable to expect that dementia will increase as a share of diabetes complications.

Constraining the diabetes epidemic could reduce dementia incidence in future decades, however the effect is likely to be gradual. The risk of CVD improves almost immediately in response to interventions that decrease serum cholesterol, BP or smoking [32]. In contrast, our study suggests a much longer time lag between decreases in population rates of obesity and diabetes, and subsequent reductions in the dementia and disability burden.

Evidence suggests that upstream, structural interventions are far more powerful prevention strategies, and also more likely to reduce inequalities, than individual level agentic interventions [33]. Several UK policies now aim to halt the rise in obesity and diabetes prevalence, including the Calorie Reduction Programme, the Sugar Reduction Programme, marketing campaigns, the Soft Drinks Industry Levy and National Diabetes Prevention Programme [11, 12, 34]. In terms of individual level interventions, the English NHS Health Check programme for adults aged 40–74, which already includes HbA_1c_ or fasting blood glucose to identify diabetes in people with high BMI and/or BP, has recently been updated to include dementia advice. This may be useful for patient education as recent research suggested nearly half of adults over 50 did not know that a healthy lifestyle could help prevent dementia [35].

### Conclusions

Future reductions in diabetes prevalence could reduce the burden of disability and dementia, however these reductions in dementia and disability burden will be observed in the mid to long term. Other preventative interventions targeting the shared determinants of these and other non-communicable diseases must be harnessed to stem the rising tide of dementia and disability in England.

## Electronic supplementary material

ESM (PDF 1960 kb)

## Data Availability

The mortality rates data used in this analysis are available at the Office for National Statistics website: www.ons.gov.uk/peoplepopulationandcommunity/populationandmigration/populationprojections/methodologies/2016basednationalpopulationprojectionsconsultationpapers. Data on prevalence of obesity and diabetes used in this analysis are available at the NHS Digital website: https://digital.nhs.uk/data-and-information/publications/statistical/health-survey-for-england/health-survey-for-england-2011-trend-tables. ELSA data used in this analysis are available at UK Data Service: 10.5255/UKDA-SN-5050-16.

## Abbreviations

CVD: Cardiovascular disease
DPM: Diabetes Prevalence Model
ELSA: English Longitudinal Study of Ageing
IMPACT-BAM: IMPACT Better Ageing Model
LYG: Life years gained
PAR: Population attributable risk
PARF: Population attributable risk fraction
PHE: Public Health England
UI: Uncertainty interval
