# Supplementary material for: Potential impact of diabetes prevention on mortality and future burden of dementia and disability: a modelling study
Source: Diabetologia. 2019 Nov 15;63(1):104–15. doi: 10.1007/s00125-019-05015-4 (PMC6890625; doi:10.1007/s00125-019-05015-4)
Supplement: Supplementary file 1 — (PDF 1960 kb) [file 125_2019_5015_MOESM1_ESM.pdf]

Electronic Supplementary Material

The potential impact of diabetes prevention on the future burden of dementia and disability

Authors:

Bandosz P[1,3]; Ahmadi-Abhari S[2,4]; Guzman M[1]; Pearson-Stuttard[1,5] J; Collins B[1]; Whittaker, H[6] Shipley, MJ[2]; Capewell S[1]; Brunner EJ*[2]; O'Flaherty M*[1].

* Joint senior authors

[1]Department of Public Health and Policy, University of Liverpool, Liverpool, United Kingdom

[2]Department Epidemiology & Public Health, University College London, London, United Kingdom

[3]Department of Prevention and Medical Education, Medical University of Gdansk, Gdansk, Poland

[4]Ageing Epidemiology (AGE) Research Unit, Imperial College London, London, United Kingdom

[5]School of Public Health, Imperial College London, London, United Kingdom

[6]National Heart and Lung Institute (NHLI), Imperial College London, London, United Kingdom

Contents

1. IMPACT-BAM model diagram 3
2. Mortality projections 3
3. Validation of the model against observed data 7
4. Policy layer 10
4.1 Basic concept 10
4.2 Literature review and Meta-analysis 11
4.2.1 Methods 11
4.2.2 Results 12
4.3 Multilevel exposure 15
4.4 Projection of future diabetes trends 17
4.5 Projecting the distribution of duration of diabetes 18
References: 23

1. IMPACT-BAM model diagram

ESM Fig. 1 presents IMPACT-BAM model structure. Detailed description of the baseline model, states definition, and calculation of transition probabilities have been previously described [1, 2].

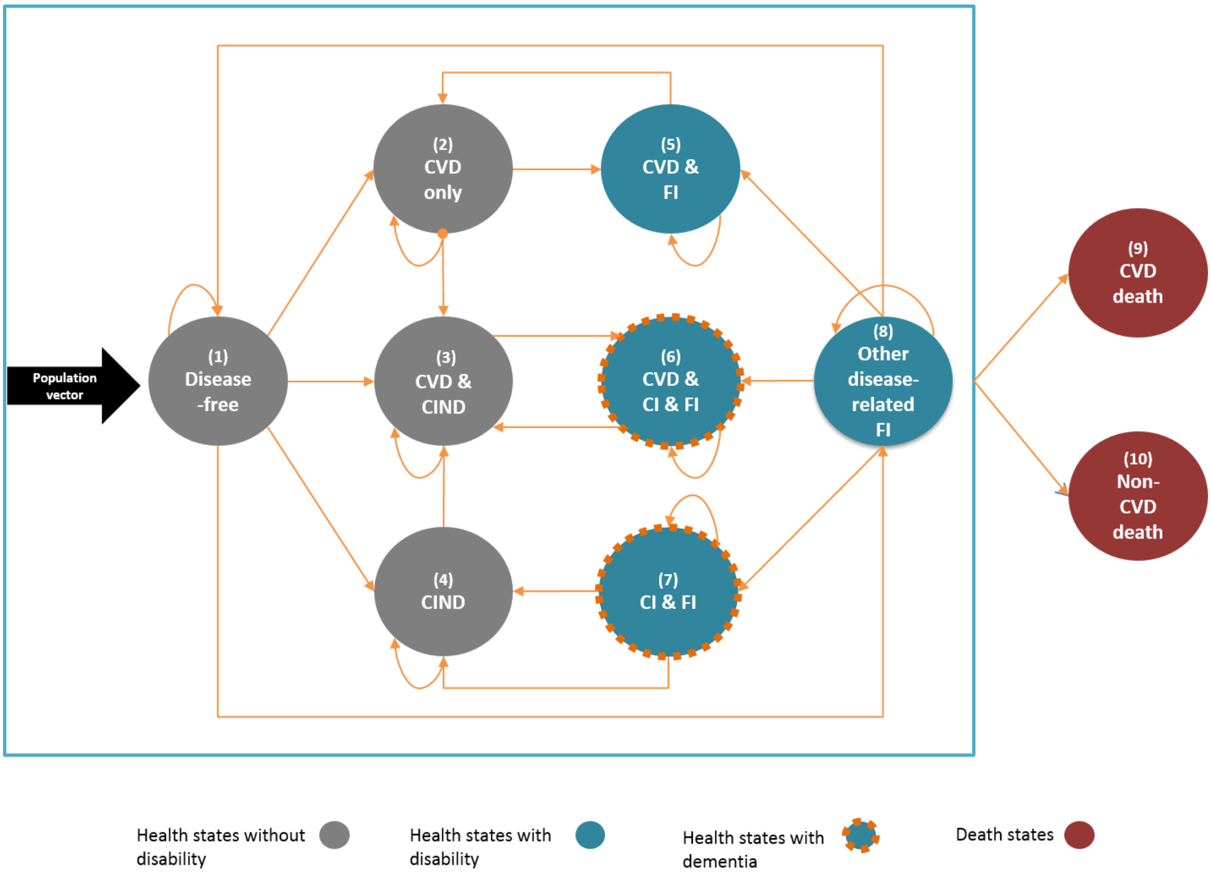

ESM Fig. 1. : IMPACT-BAM model structure. Transitions to death states 9 and 10 are possible from any state.

2. Mortality projections

For the present study, future projections for cardiovascular and non-cardiovascular mortality rates by age and sex were estimated based on observed mortality rates reported by the ONS up to 2016. P-spline smoothed lines [3-5] were fitted to logarithmic transformed CVD and non-CVD mortality rates in each 5-year age band from 1990 to 2016 by sex using the pspline function is Stata (StataCorp. 2017. Stata Statistical Software: Release 15. College Station, TX: StataCorp LLC). Change in P-spline smoothed values of log-transformed mortality rates over subsequent years followed a normal distribution. To obtain missing values for change in mortality rates in future years, linear regression models were fitted to the change in log-transformed mortality rates over subsequent years by sex with interaction terms for 5-year age groups. Linear predictions calculated from the linear regression coefficients represented predicted change in log-mortality rates for subsequent years which were used to project mortality rates to the future until 2060. For all age-groups P-spline smoothed log-transformed mortality rates declined over time but the magnitude of decline also declined, resulting in projected mortality rates reaching a plateau in the decades to come. Standard error for the linear predictor was calculated as time in years

multiplied by the standard error of the linear prediction.This method closely matches the method used for the official mortality projections used by the ONS [6]. All-cause mortality rates projected to the future using the described method closely matched all-cause mortality rate projections from the ONS (not shown)

The projected CVD and non-CVD mortality rates men and women are presented in ESM figures 2-5.

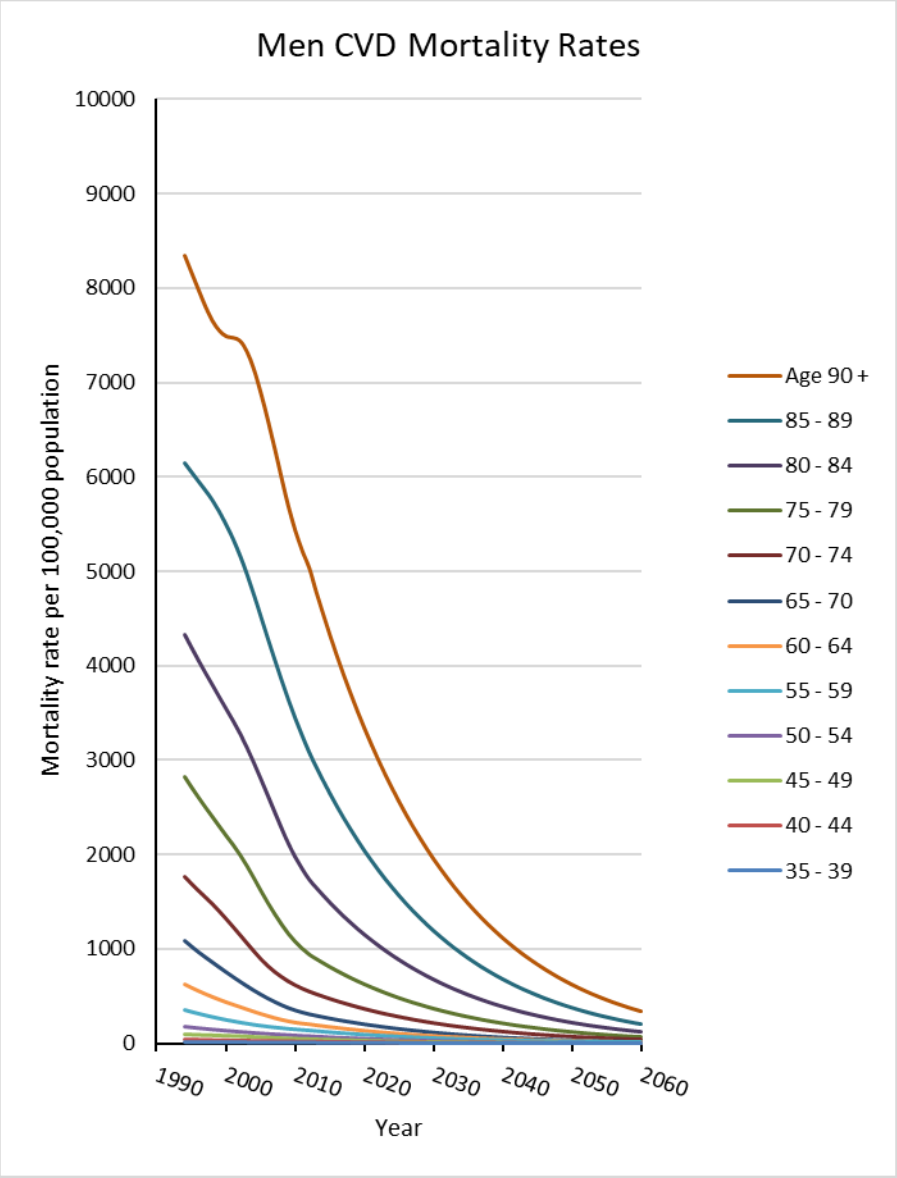

ESM Fig. 2. Projected CVD mortality rates in men

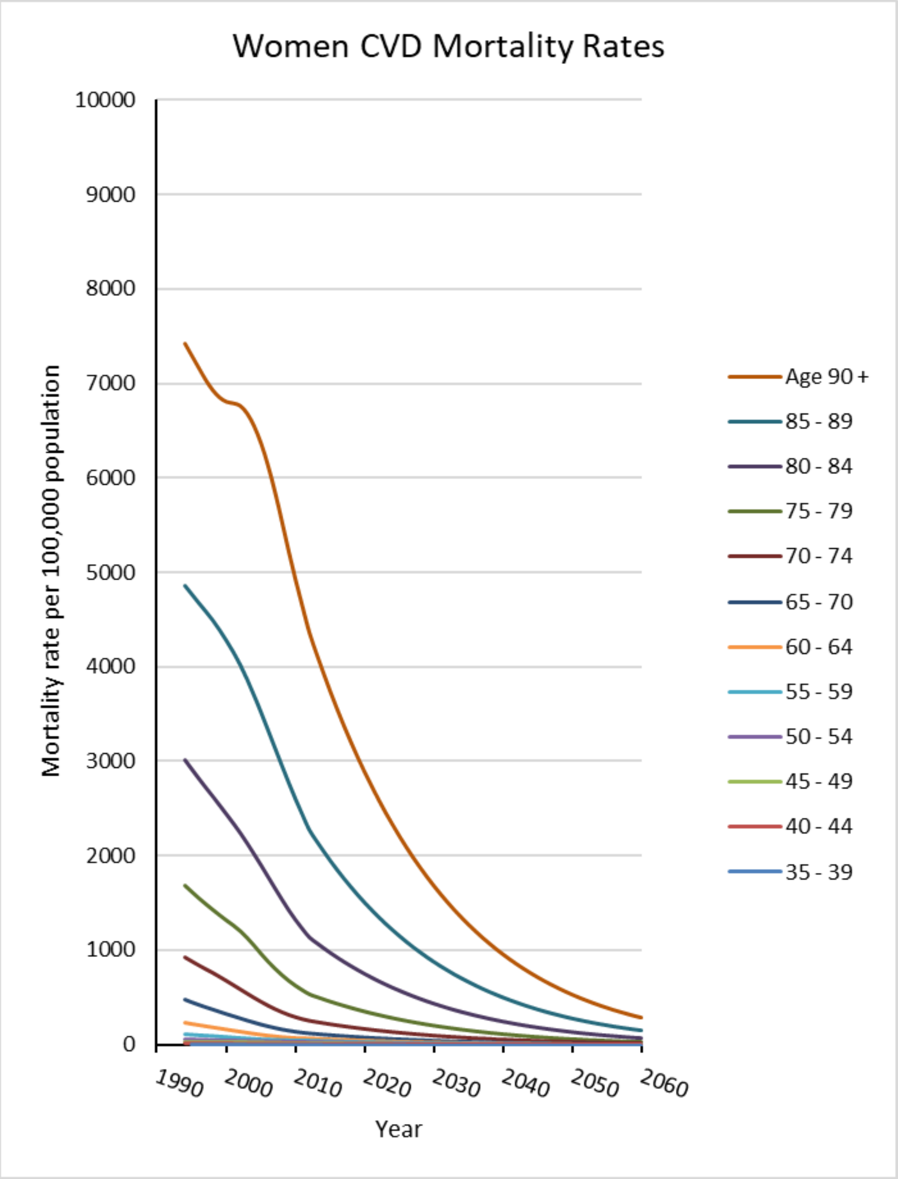

ESM Fig. 3. Projected CVD mortality rates in women

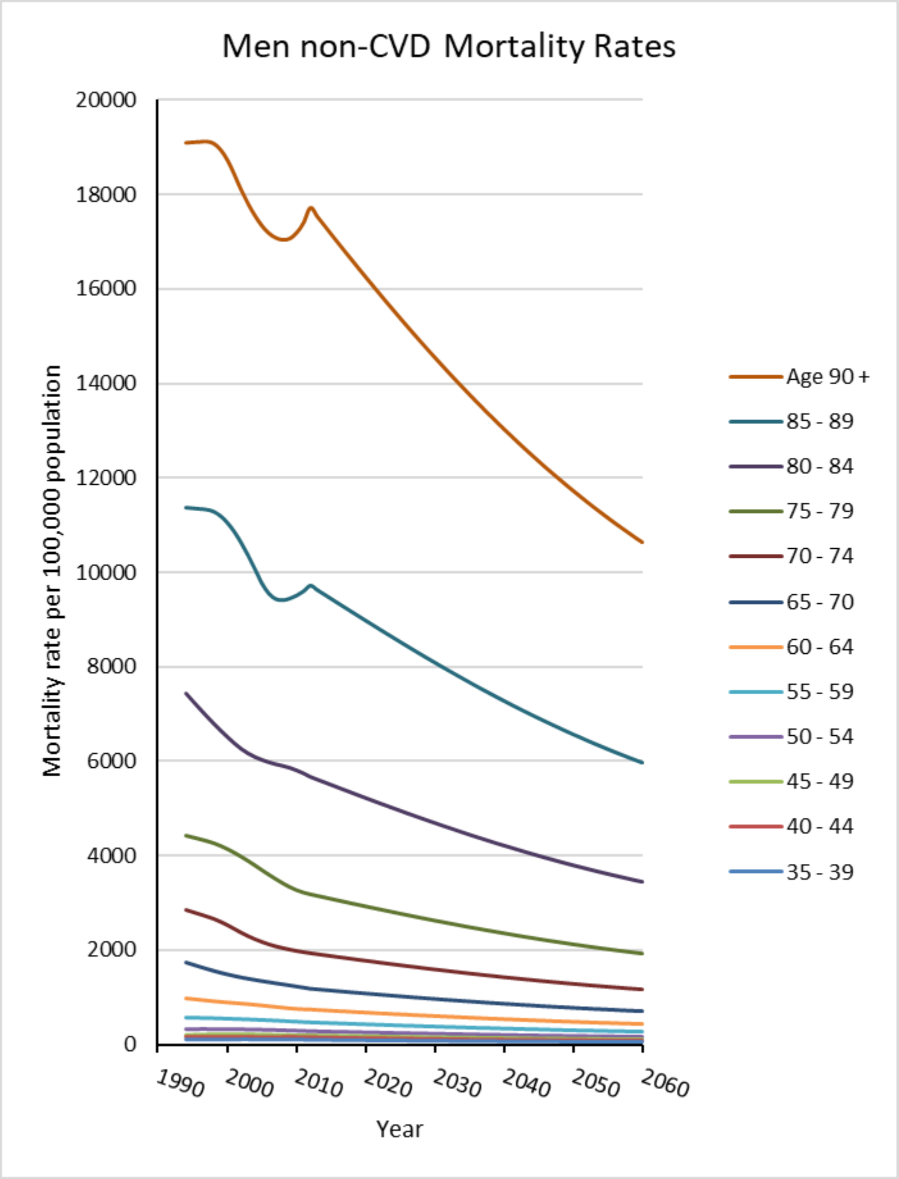

ESM Fig. 4. Projected non-CVD mortality rates in men

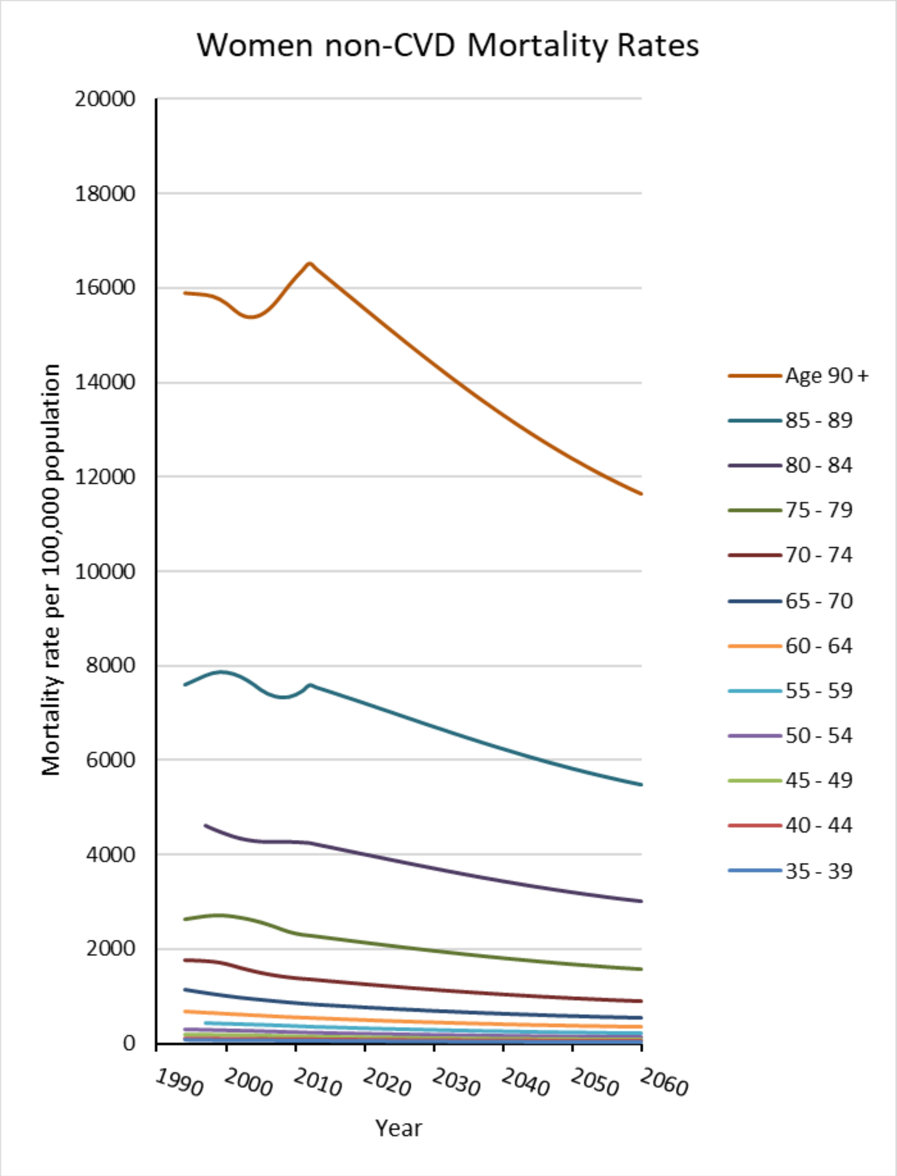

ESM Fig. 5. Projected non-CVD mortality rates in women

3. Validation of the model against observed data

We carried out partially-dependent validation of our estimates of CVD and Non-CVD deaths with observed ONS mortality data reported for England & Wales for the period 2006-2016. Using the definition suggested by the ISPOR-SMDM Modeling Good Research Practices Task Force [7], partially-dependent validation occurs when the external source to which the output is being compared to was used to build a part of the model, but it does not wholly determine the outcome to be validated. Because of above, this validation confirms internal consistency of the model rather than real-world validity of the projections. The model provided a good match to the ONS estimates of the number of CVD and Non-CVD deaths (ESM Fig. 6 and ESM Fig. 7).

We carried out independent validation (i.e. no information from these sources was used to build the model) of our model estimates of the prevalence of CVD and dementia. Our estimates of CVD

in 2011 for men fall within the 95% confidence intervals reported by the HSE [8]. However, our model estimates a slightly higher prevalence of CVD in women (ESM Fig. 8).

Our age-specific estimates of dementia prevalence in 2011 were akin to those reported in CFAS II for the same year (ESM Fig. 9). Most of our estimates of age-specific prevalence fall within the 95% confidence interval reported by CFAS II. The only exceptions were for women 85+, where our estimates were lower than those from CFAS.

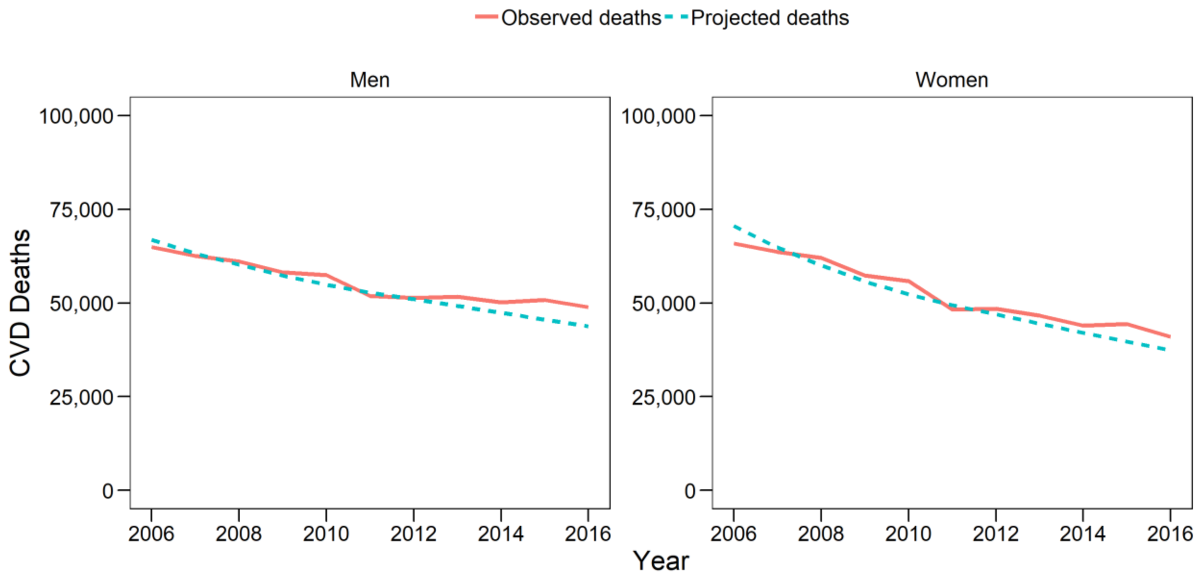

ESM Fig. 6. Predicted CVD mortality in England & Wales against ONS estimates 2006-2016

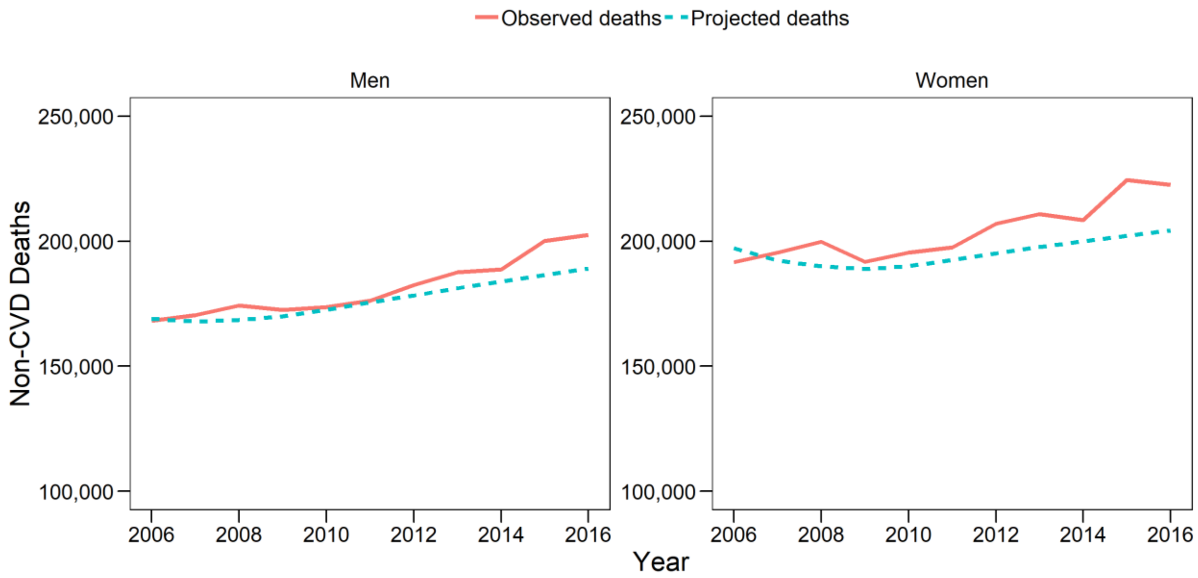

ESM Fig. 7. Predicted Non-CVD mortality in England & Wales against ONS estimates 2006-2016

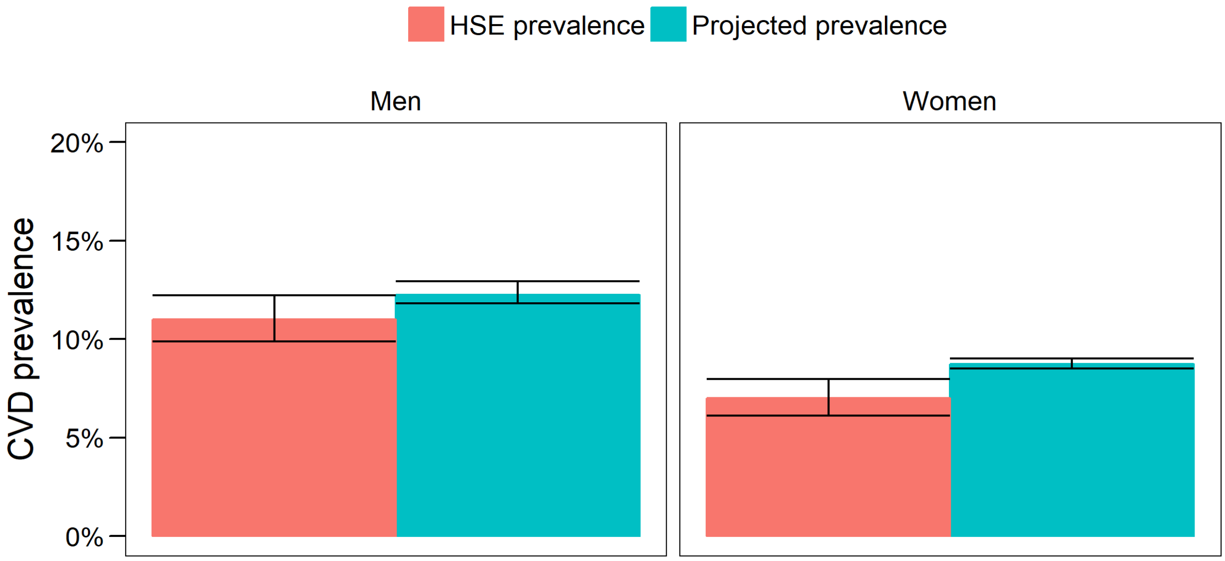

ESM Fig. 8. Predicted prevalence of CVD against Health Survey for England estimates in 2011. The error bars represent 95% uncertainty intervals for IMPACT-BAM predictions and 95% confidence intervals for HSE estimates.

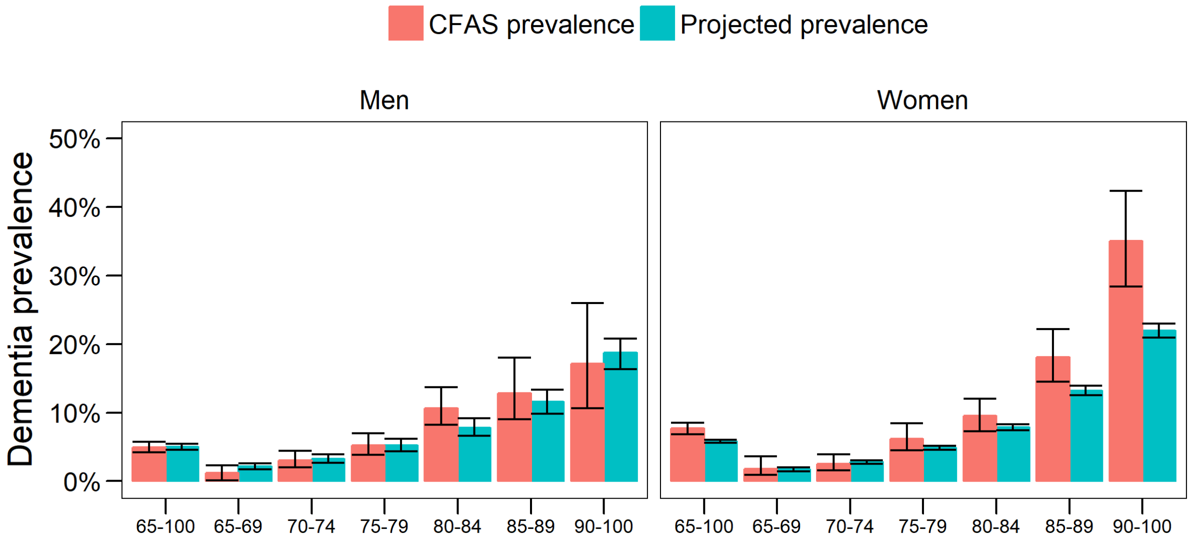

ESM Fig. 9 Age and gender-specific predicted the prevalence of dementia against CFAS estimates in 2011. The error bars represent 95% uncertainty intervals for IMPACT-BAM predictions, and 95% confidence intervals for CFAS II estimates.

4. Policy layer

This latest version of IMPACT-BAM evaluates the impact of changes in risk factors at the population level (due to hypothetical policy interventions) on future cases of dementia, disability, CVD and mortality.

4.1 Basic concept

We modified relevant transition probabilities in the baseline model according to assumed changes in specific risk factors using a population attributable risk fraction (PARF) approach. The PARF calculates the proportion by which disease burden would be reduced if the prevalence of a risk factor was reduced to zero. Symbolically, PARF is the following[6]:

$$PARF = \frac{P \times (RR - 1)}{1 + P \times (RR - 1)}$$

Where P is the diabetes prevalence, and RR is the disease risk ratio. P and RR are age and sex-specific. In this paper, we are interested in how this PARF varies because of changes in diabetes prevalence. Symbolically, this would be:

$$\Delta PARF = \frac{(P - P') \times (RR - 1)}{1 + P \times (RR - 1)}$$

Where P' is the prevalence of the disease after the intervention. This equation is equivalent to the potential impact fraction (PIF) equation for discrete risks factors generally reported in publications [9].

To obtain the RRs describing the association between diabetes and incidence of dementia, the incidence of recovery from functional impairment, CVD incidence/mortality and non-CVD mortality, we conducted a systematic review and meta-analyses (See section 4.2). The RRs obtained from these meta-analyses were then adjusted by the duration of diabetes (see section 4.3)

4.2 Literature review and Meta-analysis

4.2.1 Methods

PubMed was searched using the following search strategy to identify studies reporting the association between diabetes and the incidence of dementia or incidence of recovery from functional impairment:

Dementia:

diabetes[Title/Abstract]

AND (**cognitive impairment**[Title/Abstract] OR **mild cognitive impairment**[Title/Abstract] OR **dementia**[Title/Abstract] OR **Alzheimer's disease**[Title/Abstract])

AND (**cohort**[Title/Abstract] OR **longitudinal**[Title/Abstract] OR **prospective**[Title/Abstract] OR **follow up**[Title/Abstract] OR **clinical trial** [Title/Abstract])

AND (**risk**[Title/Abstract] OR **incidence**[Title/Abstract] OR **ratio**[Title/Abstract])

AND **Humans**[Mesh] AND **English**[lang]

Functional Impairment:

diabetes[Title/Abstract]

AND (**functional impairment**[Title/Abstract] OR **basic activities of daily living**[Title/Abstract] OR **activities of daily living**[Title/Abstract] OR **ADL**[Title/Abstract] OR **disability**[Title/Abstract] OR **frailty**[Title/Abstract])

AND (**cohort**[Title/Abstract] OR **longitudinal**[Title/Abstract] OR **prospective**[Title/Abstract] OR **follow up**[Title/Abstract] OR **clinical trial** [Title/Abstract])

AND (**risk**[Title/Abstract] OR **incidence**[Title/Abstract] OR **ratio**[Title/Abstract] OR **recovery** [Title/Abstract])

AND **Humans**[Mesh] AND **English**[lang]

References for retrieved relevant publications were hand searched for any papers that may have been excluded from the PubMed search. Studies were included in the meta-analysis if they were prospective, cohort, or longitudinal studies; published in English; conducted in Europe, North America, or Australia; dementia was ascertained using DSM III, DSM-IV, or NINDES-AIRNEN criteria; disability/functional impairment was ascertained by impairment in independently conducting one or more basic activities of daily living (getting in or out of bed, cutting food and eating, using the toilet, bathing/showering, putting on clothes including shoes and socks, walking across the room) and the study reported the relative risk or hazard ratio of incident dementia or incidence functional impairment in individuals with diabetes compared to those without. The relative risk or hazard ratio with the maximum level of adjustment was included in the meta-analysis. Studies were excluded if the follow up ended before 1990 or if an updated version of the study was later published.

Meta-analyses were conducted using the metan function of the STATA software, version 15, to obtain a pooled estimate of the association between diabetes and dementia or functional impairment. Study weights in the meta-analysis were assigned in proportion to the person-years of follow up in each study. Heterogeneity between studies was assessed using the I2 statistic. Although the I2 statistic was high for the meta-analysis related to the dementia outcome, there was not considerable heterogeneity in terms of the design and quality of the studies, method for assessment of exposure or outcome, ethnicity, age and sex structure, or the results obtained. A fixed effects meta-analysis was thus conducted for both outcomes. The difference between a fixed versus random effects meta-analysis on the pooled estimate was small.

4.2.2 Results

Dementia

The search strategy returned 732 titles published by December 2017. Sixty-three titles were found to be relevant, including two studies identified by hand searching the references, and the full texts were examined by two independent reviewers (HW and SAA). Twenty-two studies met the inclusion and exclusion criteria and were included in the meta-analysis[10-31].

The measures of association in all studies were adjusted for age, sex and education or socioeconomic status. Several studies additionally adjusted for the confounding effects of body mass index (BMI) and other known risk factors [12, 14, 17, 19, 23-29].

The results of the meta-analysis are presented in ESM Fig. 10. In a meta-analysis of all 22 studies, the pooled hazard ratio of dementia associated with diabetes was 1.45 (95% CI 1.27, 1.62). The pooled HR remained unchanged (1.47 (95% CI 1.27, 1.64) in a meta-analysis of the 12 studies that had adjusted for BMI or waist circumference [12, 14, 17, 19, 23-29].

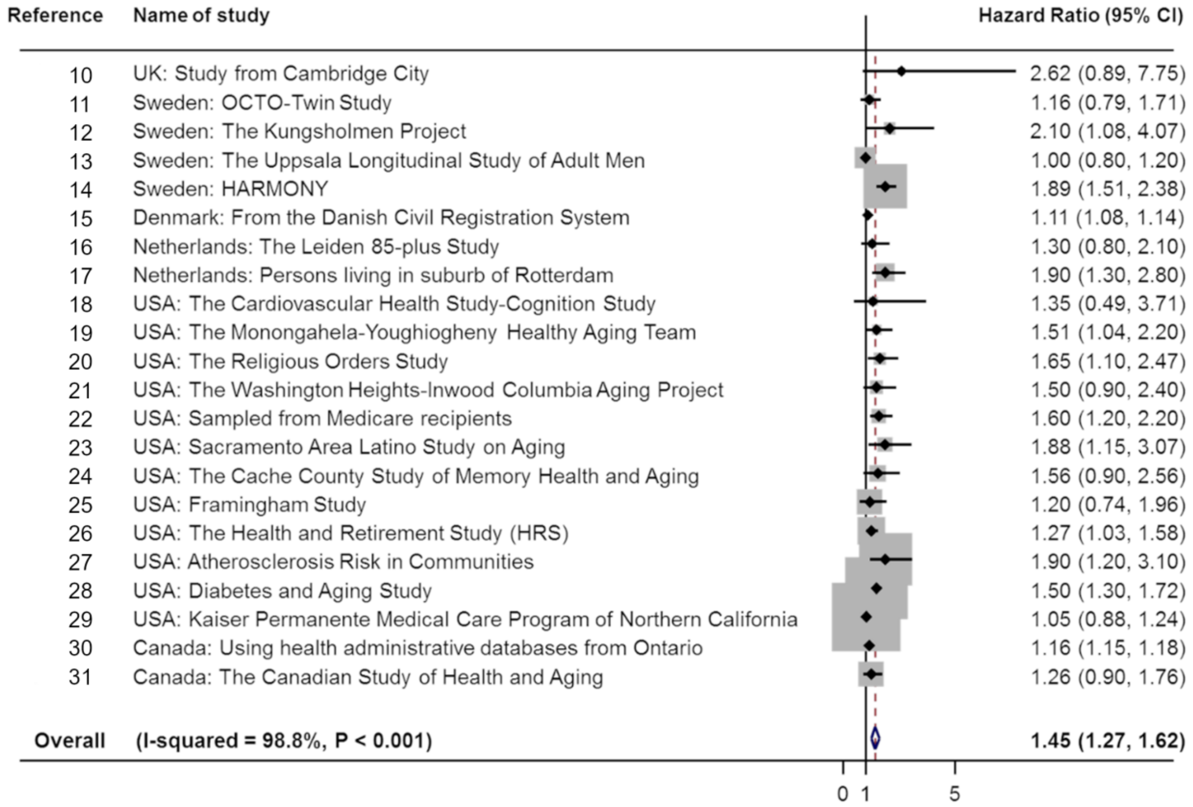

ESM Fig. 10. Forest plot is summarising studies investigating the association of diabetes and incident dementia.

Functional impairment

The search strategy returned 521 titles published by December 2017. Twenty-six titles were found to be relevant, and the full texts were examined by two independent reviewers (HW and SAA). Twelve studies met the inclusion and exclusion criteria and were included in the meta-analysis [32-43]. The measures of association in all studies were adjusted for age, sex and education or socioeconomic status. Several studies additionally adjusted for the confounding effects of body mass index (BMI) and other known risk factors [35-38, 40, 42]. The results of the meta-analysis are presented in ESM Fig. 11. The pooled relative risk of functional impairment associated with diabetes was 1.46 (95% CI 1.33, 1.90) in a meta-analysis of all 12 studies. Among studies that additionally adjusted for BMI, the pooled relative risk was 1.52 (95% CI 1.30, 1.74).

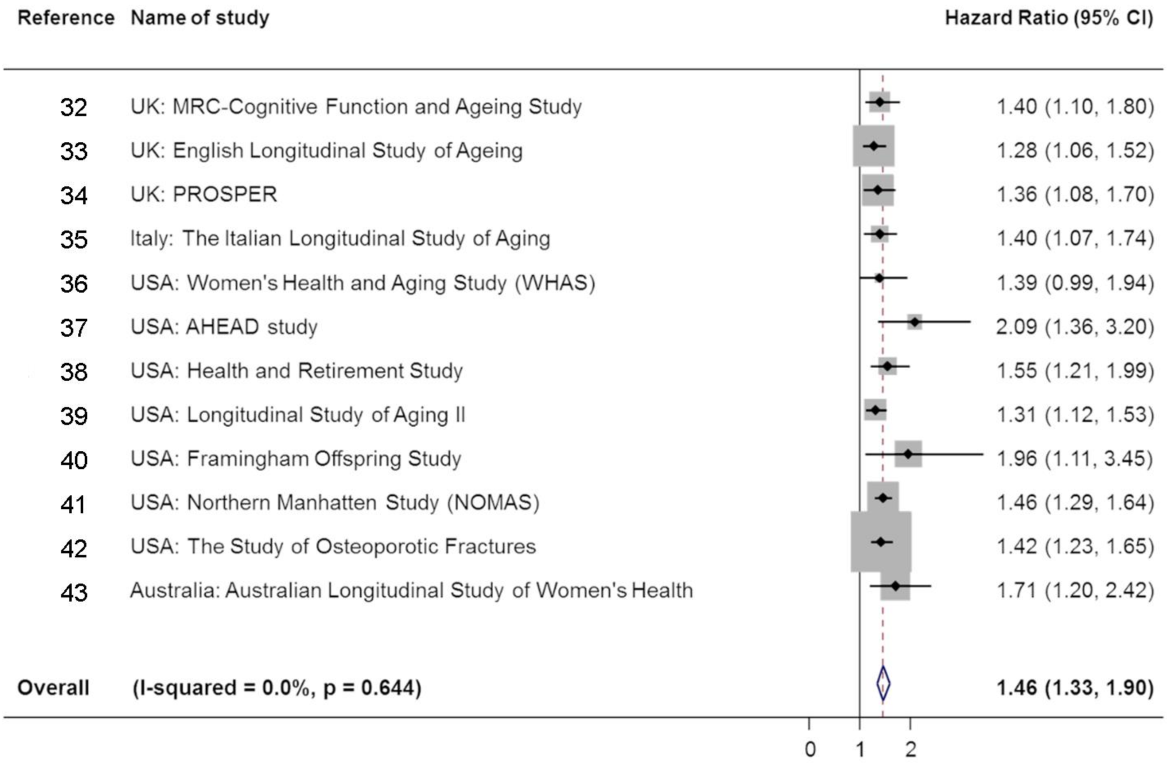

ESM Fig. 11. Forest plot is summarising studies investigating the association of diabetes and functional impairment in independently conducting one or more activities of daily living.

Recovery from functional impairment

One study (UK: English Longitudinal Study of Ageing) was identified that reported the RR of recovery from functional impairment comparing individuals with and without diabetes (RR of recovery 0.93 (95% CI 0.86-1.00)) [33].

CVD incidence / CVD Mortality

The Emerging Risk Factors Collaboration reported individual-level meta-analysis for 698 782 people (52 765 non-fatal or fatal vascular outcomes; over 8·49 million person-years of follow up) from 102 prospective studies [44]. The Pooled RR for Non-fatal myocardial infarction in individuals with diabetes compared to those without was 1.82 (95% CI 1.64-2.03). The corresponding figure for death from coronary heart disease was 2.31 (95% CI 2.05-2.60), similar to the pooled RR of vascular deaths (2.32 (95% CI, 2.11 to 2.56)) reported in a separate meta-analysis [45].

Non-CVD Mortality

The Emerging Risk Factors Collaboration reported an individual level data meta-analysis on the association of diabetes with cause-specific deaths among 820,900 individuals over a total of 12.3 million person-years of follow up in 97 prospective studies [45]. The pooled adjusted HR was 1.25 (95% CI, 1.19 to 1.31) for death from cancer, and 1.73 (95% CI, 1.62 to 1.85) for death from nonvascular causes not attributed to cancer. Assuming ~30% of deaths are from CVD causes,

~40% from cancer, and ~30% from non-cancer non-CVD causes, a meta-analysis (with weights in proportion to numbers of death from each cause) to obtain a pooled HR for non-CVD mortality associated with diabetes was 1.46 (95% CI 1.40, 1.52). To check the internal validity of this estimate, a meta-analysis was performed to pool the HR obtained for non-CVD mortality with that for CVD mortality to obtain the pooled HR for all-cause mortality [46]. This estimate was compared, and was consistent, with the HR for all-cause mortality obtained from the individual level data meta-analysis (1.74 (95% CI 1.64, 1.84) vs 1.80 (95% CI 1.71, 1.90) [45].

4.3 Multilevel exposure

There is evidence suggesting the risk of unfavourable outcomes of diabetes strongly depends on the duration of the disease. For example, ADVANCE study reported increase in risk among diabetic subjects with the longer duration of diabetes: [47]

- for macrovascular events: 17% (12%-22%) for each 5-years of diabetes duration
- for microvascular events: 31% (26%-36%) for each 5-years of diabetes duration
- for all-cause death: 21% (15%-26%) for each 5-years of diabetes duration

Therefore, in IMPACT-BAM, we treated diabetes as a multilevel exposure risk factor, using different categories of diabetes duration as different exposure levels. We explained how we project diabetes prevalence and duration the multilevel PARF formula in section 4.4. The previous PARF equation in section 4.1 considers only two levels of risk factor exposure (exposure =0 and exposure ≠0). Thus, we used the following extension to consider multilevel exposure[5]:

$$PARF = \frac{\sum_{i=0}^{k} P_i \times (RR_i - 1)}{1 + \sum_{i=0}^{k} P_i \times (RR_i - 1)}$$

Subscript *i* refers to the *ith* exposure level. P_i=prevalence of the risk factor in *ith* exposure level, RRj= relative risk comparing *ith* exposure level with unexposed group. P_i and RR_i are age and sex-specific.

Then, $\Delta PARF$ is

$$\Delta PARF = \frac{\sum_{i=0}^{k} P_i \times (RR_i - 1)}{1 + \sum_{i=0}^{k} P_i \times (RR_i - 1)} - \frac{\sum_{i=0}^{k} P_i' \times (RR_i - 1)}{1 + \sum_{i=0}^{k} P_i \times (RR_i - 1)}$$

The RRs obtained from our literature review and meta-analyses described in the previous section were not stratified by diabetes duration. Therefore, to account for diabetes duration, we corrected RRs for longer than 5 years duration of diabetes by expected increase in risk based on data from ADVANCE (see above). This assumption might result in overestimation of the effect size.

The following table (ESM Table 1) reports the unadjusted RRs from our literature review and meta-analyses and the adjusted relative risks values used in the PARF approach.

ESM Table 1. Transition probabilities affected by the change in diabetes prevalence:

Risk	TPs affected	RRs from literature review and meta-analyses	Assumed increase in risk for each 5-year of diabetes duration.
CVD mortality	P1_8,<br>P2_8,<br>P3_8,<br>P4_8,<br>P5_8,<br>P6_8,<br>P7_8,<br>P10_8,	2.32 (95% CI, 2.11 to 2.56)	21% (95%CI: 15% to 26%)
Non-CVD mortality	P1_9,<br>P2_9,<br>P3_9,<br>P4_9,<br>P5_9,<br>P6_9,<br>P7_9,<br>P10_9,	1.46 (95% CI 1.40, 1.52)	21% (95%CI: 15% to 26%)
CVD incidence	P1_2,<br>P4_3	1.82 (95% CI 1.64-2.03)	17% (95%CI: 12%-22%)
Dementia incidence	P1_3, P1_4,	1.42 (95% CI 1.26 – 1.59)	17% (95%CI: 12%-22%)
	P2_3	1.46 (95% CI 1.33 – 1.59)	17% (95%CI: 12%-22%)
Disability incidence	P2_5,	1.46 (95% CI 1.33 – 1.59)	17% (95%CI: 12%-22%)
	P3_6,<br>P1_10	1.46 (95% CI 1.33 – 1.59)	Average of 17% (95%CI: 12%-22%) and 31% (95%CI: 26% to 36%
	P4_7,	1.46 (95% CI 1.33 – 1.59)	31% (95%CI: 26% to 36%)
Disability reversal	P5_2, P6_3, P7_4, P10_1	0.93 (95% CI 0.86-1.0)	No-adjustment

4.4 Projection of future diabetes trends

We evaluated three potential future scenarios of trends in diabetes duration and compared them to a baseline scenario which assumes the continuation of the current obesity trend. Increasing prevalence of diabetes is mostly driven by the obesity epidemic. To obtain reasonable scenarios of possible future trends in diabetes, we calculated the expected change in diabetes prevalence due to possible changes in the obesity trend in England.

We used the already existing Diabetes Prevalence Model, published by Public Health England to translate changes in obesity into trends in diabetes prevalence [48].

The baseline scenario assumes the continuation of current obesity trends at the rate of 1% per 5 years, and the corresponding expected relative increase in diabetes prevalence is 26% between 2015 and 2060. The other scenarios assume:

1. Acceleration of obesity with the trend increasing to 5% per 5 years, increasing diabetes prevalence by 49% between 2015 and 2060
2. Halt to any further increase in obesity, resulting in a slower increase in diabetes prevalence of 20% between 2015 and 2060
3. Reversing current obesity trend (decrease obesity at 3% per 5 years); this will not immediately decrease the prevalence of diabetes, resulting in a relative increase in diabetes prevalence of 7% between 2015 and 2060 (see ESM Fig. 12).

The projection of diabetes prevalence trend was then smoothed to obtain diabetes prevalence values stratified by single year. As Diabetes Prevalence Model allowed to forecast up to 2035, We extended the projections until 2060 using local polynomial regression (loess() function of R package, with span parameter = 10 and degree of polynomials = 2). The result of this process is shown in ESM Fig. 12 and manuscript Table 1:

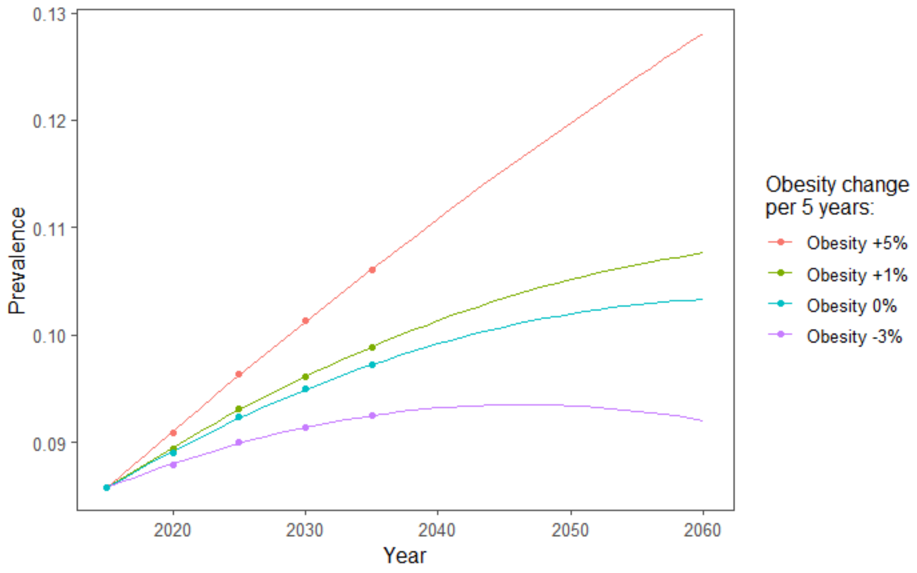

ESM Fig. 12. Projection of future diabetes prevalence in England under different assumptions of future obesity changes, according to DPM.

PHE model does not allow to project diabetes prevalence stratified by age and sex. We used then age/sex gradient for the prevalence of diabetes from another source, assuming this gradient will be similar across calendar years of the projections. A Canadian study data was used to obtain this gradient since no English national studies known to authors reported diabetes prevalence for older age groups [49]. Then the distribution was smoothed across age groups to obtain single age-stratified values.

4.5 Projecting the distribution of duration of diabetes

Notice that the extended PARF formula also requires age and sex-specific estimates of the risk factor prevalence at each exposure level. For any given year, sex and age, the number of diabetics is composed of individuals with different time spans living with the disease. For example, the overall prevalence of diabetic men aged 60 in 2040 is composed of individuals who have lived with diabetes less than five years, 5-9 years, 10-14 years, 15-19 years, 20-24 years and more than 25 years.

We used the 2014 Health Survey for England (HSE) data to obtain age and sex-specific distributions of diabetes prevalence across these six categories of diabetes duration.

For the baseline scenario, we assumed that the prevalence in each of the categories would remain constant in the future. For example, that the prevalence of diabetic men aged 60 in 2040 with a diabetes duration of 10 years is equal to the prevalence of diabetic men aged 60 in 2014 (HSE data) with a diabetes duration of 10 years.

For the scenarios, we assumed that as the result of changes in prevalence of obesity, there would be an "excess" of cases of diabetes compared to the baseline. This is visualized on the ESM Fig. 13 as the shaded area between red and black curves. These new cases will propagate across time following the ageing of their cohort. For example, the prevalence of diabetics among men aged 60 in 2040 living with the disease for more than 25 years is equal to the new cases diabetes in men aged 35 in 2015. The prevalence of diabetics among men aged 60 in 2040 living with the disease for 20 years is equal to the new cases of diabetes in men aged 40 in 2020, and so on. Formally, this is:

$$P_{s,a,t,d} = New\_cases_{s,a-d,t-d}$$

Where $P_{s,a,t,d}$ is the prevalence of diabetics sex *s*, age *a*, in year *t* and with *d* years living with the disease. To calculate the new cases, we calculate first the difference between the scenario-specific diabetes prevalence and the baseline diabetes prevalence by sex, age and calendar year: $D_{s,a,t}$. Then we assumed that new cases are equal to:

$$New\_cases_{s,a-d,t-d} = D_{s,a-d,t-d} - D_{s,(a-d-5),(t-d-5)}$$

The following figures present a graphical representation of the propagation of new cases across time: ESM Fig. 14: the new cases of diabetes in 2015 will become the new cases of diabetes of duration >25 years after 25 years (in the years 2040-2060) in the same cohort. This is done separately for every single cohort. ESM Fig. 15: the new cases of diabetes in 2015 will become the new cases of diabetes of duration 20-24 years after 20 years (in the years 2035-2039). ESM Fig. 16: the new cases of diabetes in 2015 will become the new cases of diabetes of duration 15-29 after 15 years (in the years 2030-2034). ESM Fig. 17 and ESM Fig. 18: Similar steps are performed for the duration of diabetes 10-14 and 5-9 years.

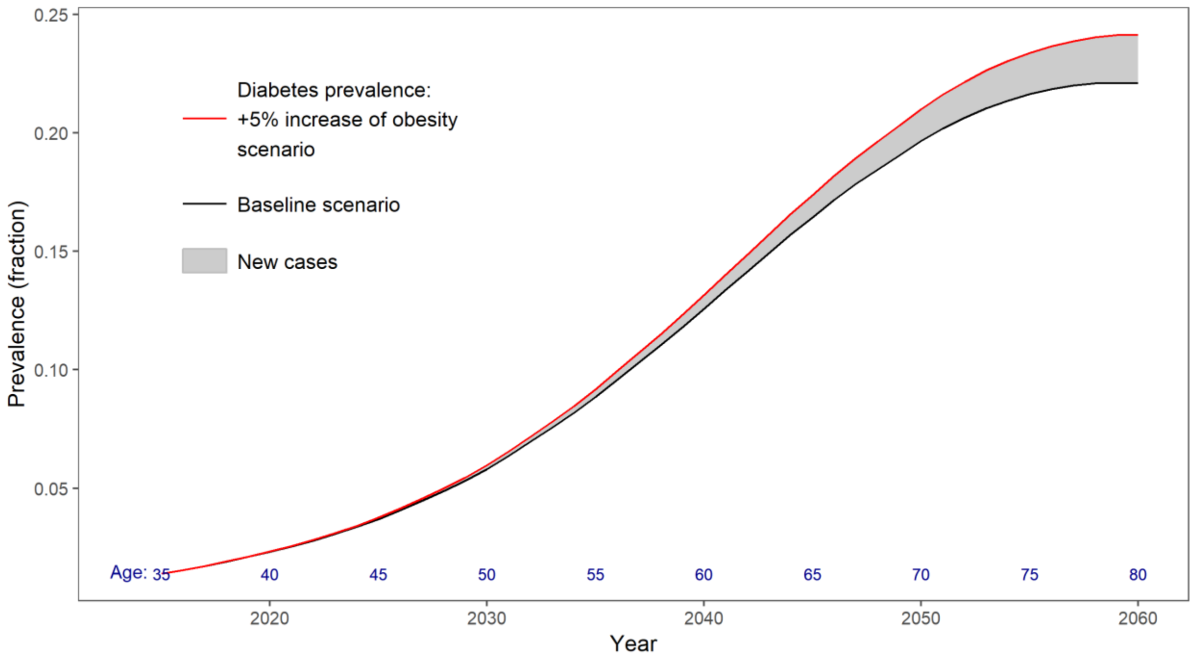

ESM Fig. 13. Prevalence of diabetes in cohort of subjects aged 35 years in 2015 for baseline sceanario and scenario assuming 5% increase in obesity per 5 years. The shaded area between red and black lines corresponds to the number of new cases of diabetes due to increase in obesity.

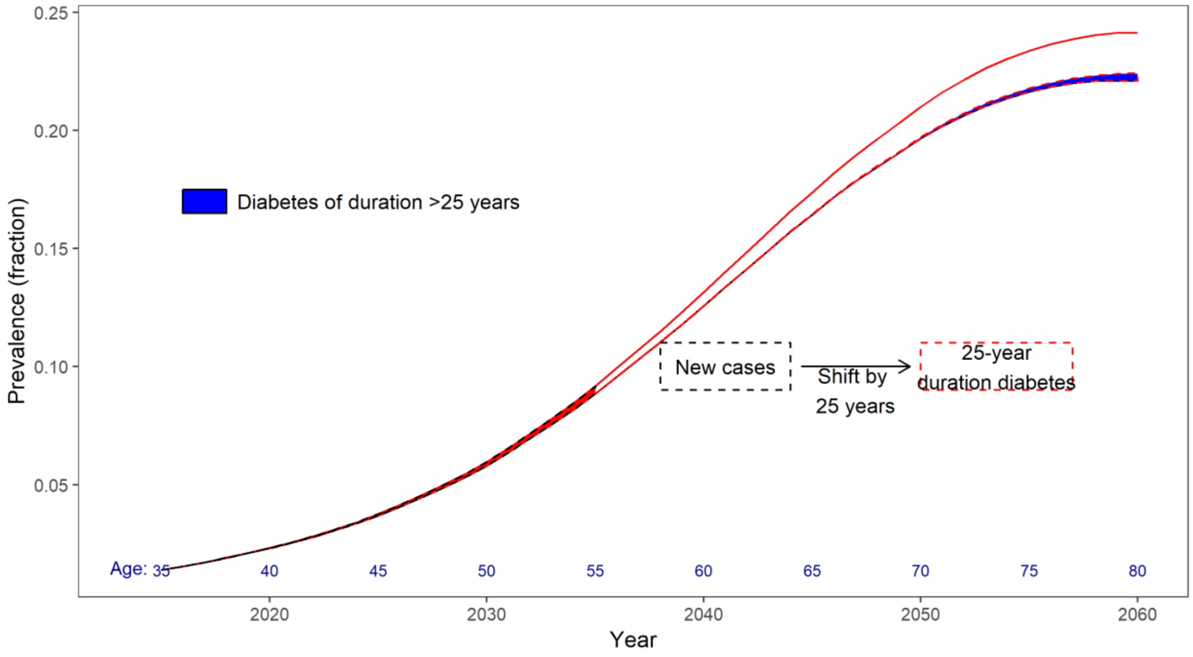

ESM Fig. 14. Example of propagation of new diabetes cases over time in cohort of subject aged 35 in 2015. New cases of diabetes become 25-year duration of diabetes after 25-year time period

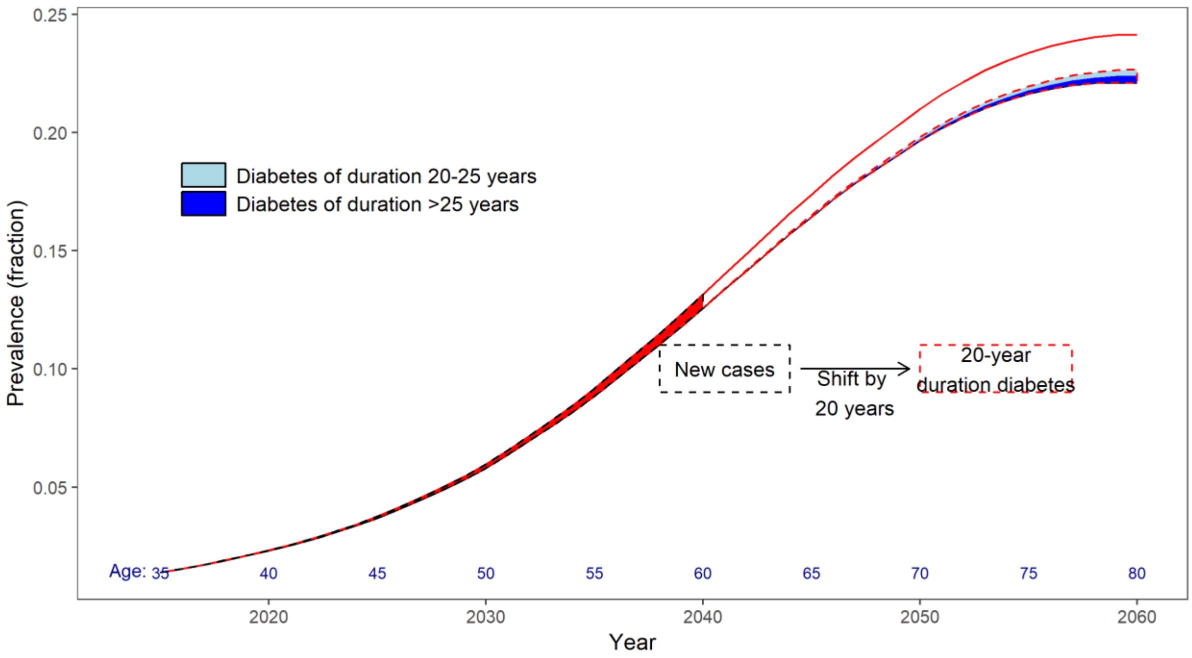

ESM Fig. 15. Example of propagation of new diabetes cases over time in cohort of subject aged 35 in 2015. New cases of diabetes become 20-year duration of diabetes after 20-year time period.

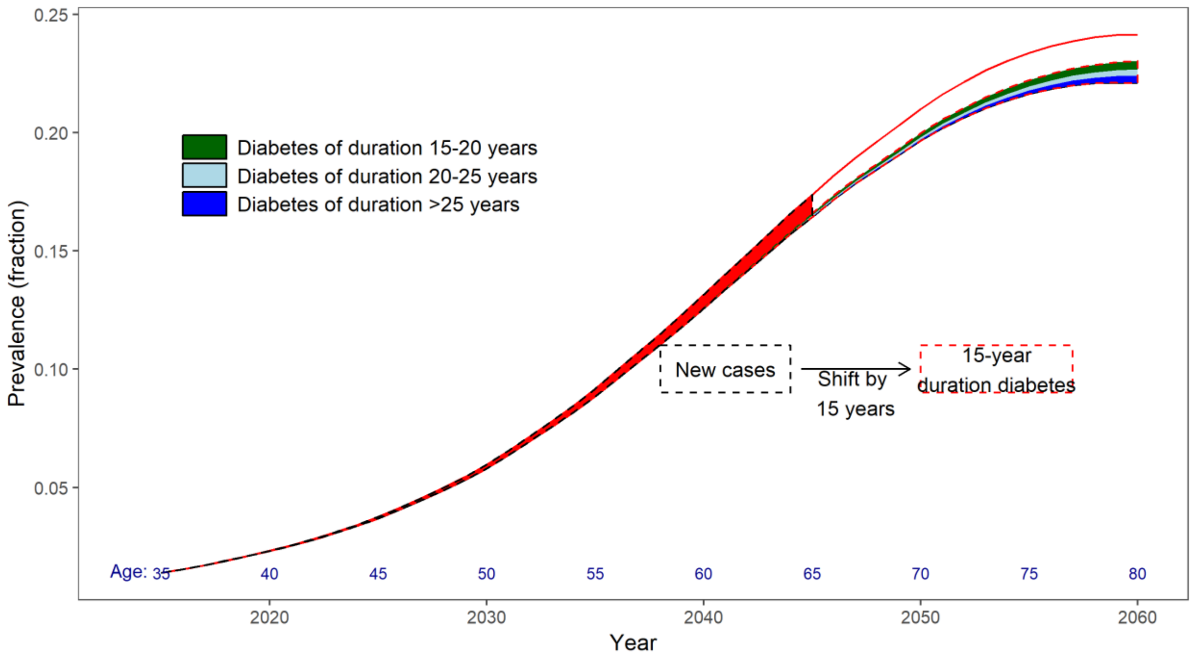

ESM Fig. 16. Example of propagation of new diabetes cases over time in cohort of subject aged 35 in 2015. New cases of diabetes become 15-year duration of diabetes after 15-year time period.

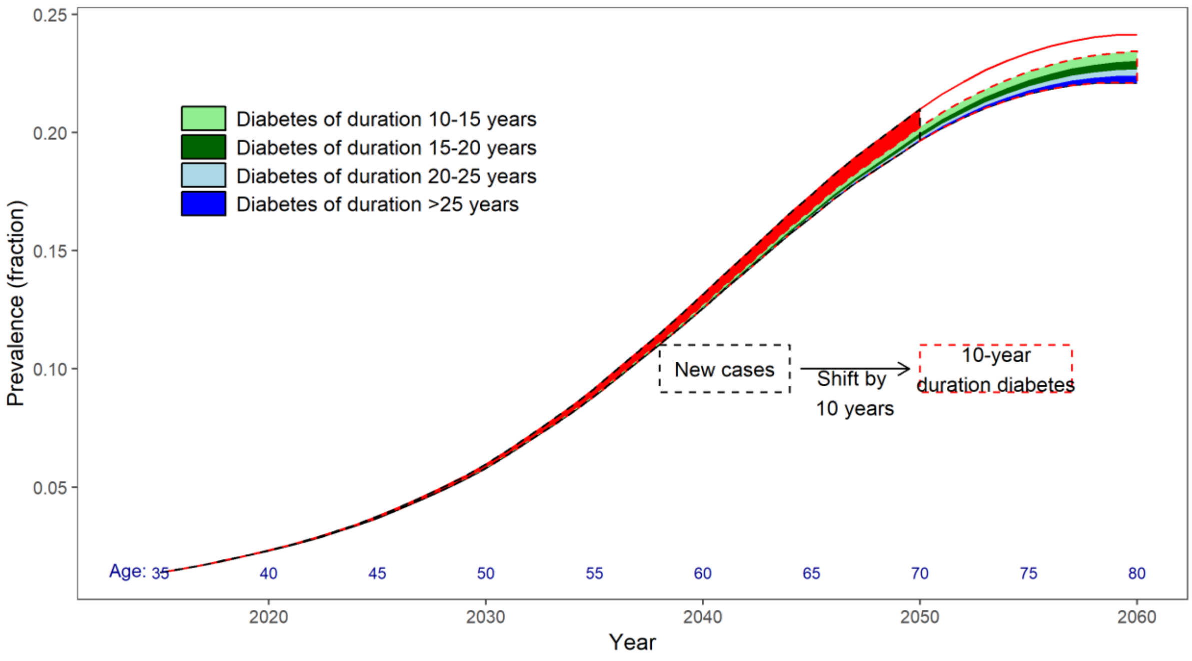

ESM Fig. 17. Example of propagation of new diabetes cases over time in cohort of subject aged 35 in 2015. New cases of diabetes become 10-year duration of diabetes after 10-year time period.

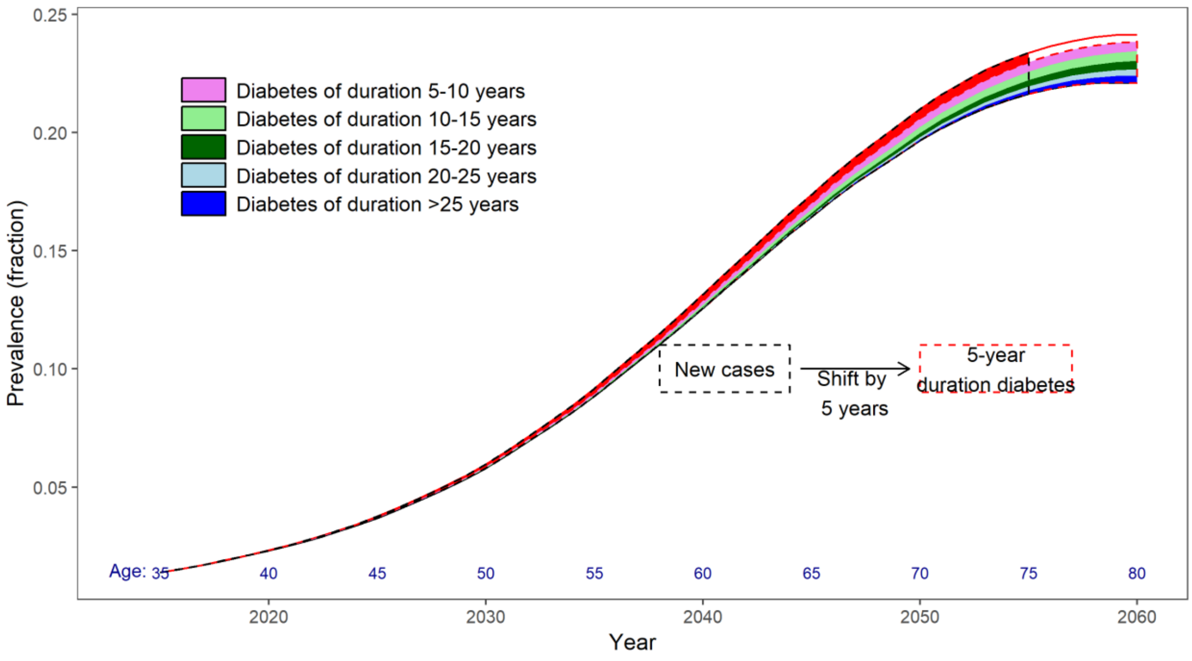

ESM Fig. 18. Example of propagation of new diabetes cases over time in cohort of subject aged 35 in 2015. New cases of diabetes become 5-year duration of diabetes after 5-year time period

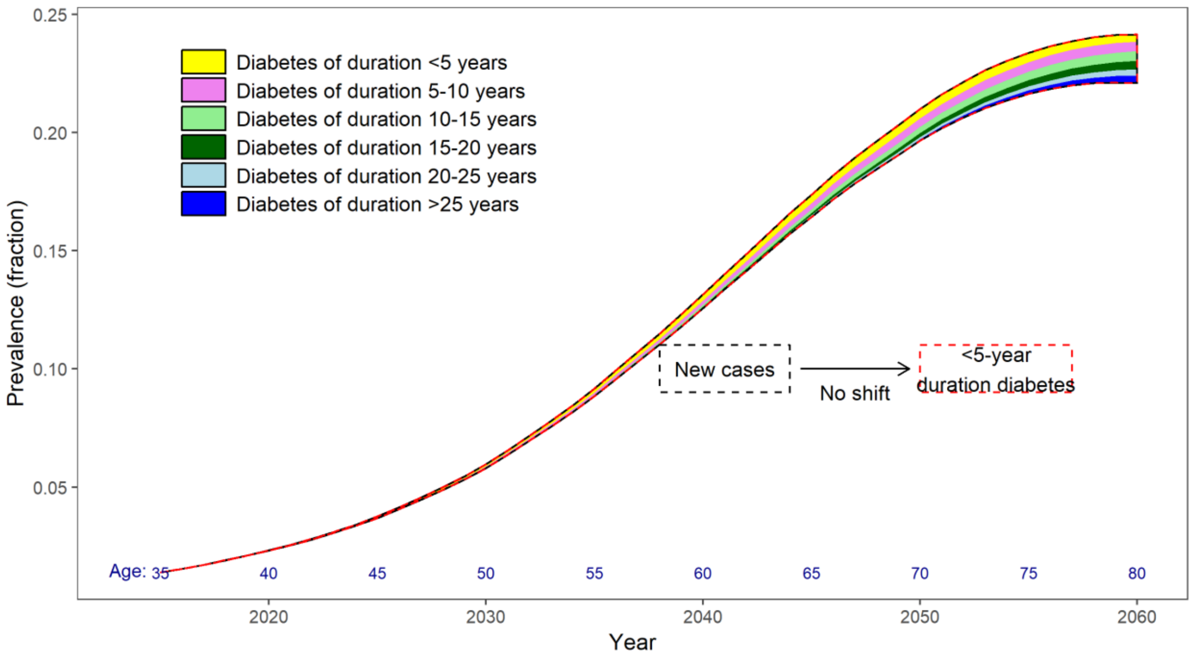

ESM Fig. 19. Example of propagation of new diabetes cases over time in cohort of subject aged 35 in 2015. Diabetes cases of duration <5 years represent the rest of total diabetes "excess" due to increase in obesity.

ESM Fig. 20 shows the final distribution of men with different duration of diabetes for the baseline scenario and the scenario assuming an increase in obesity by 5% per 5 years. These distributions were then used to calculate the multilevel exposure PARF detailed in section 4.3

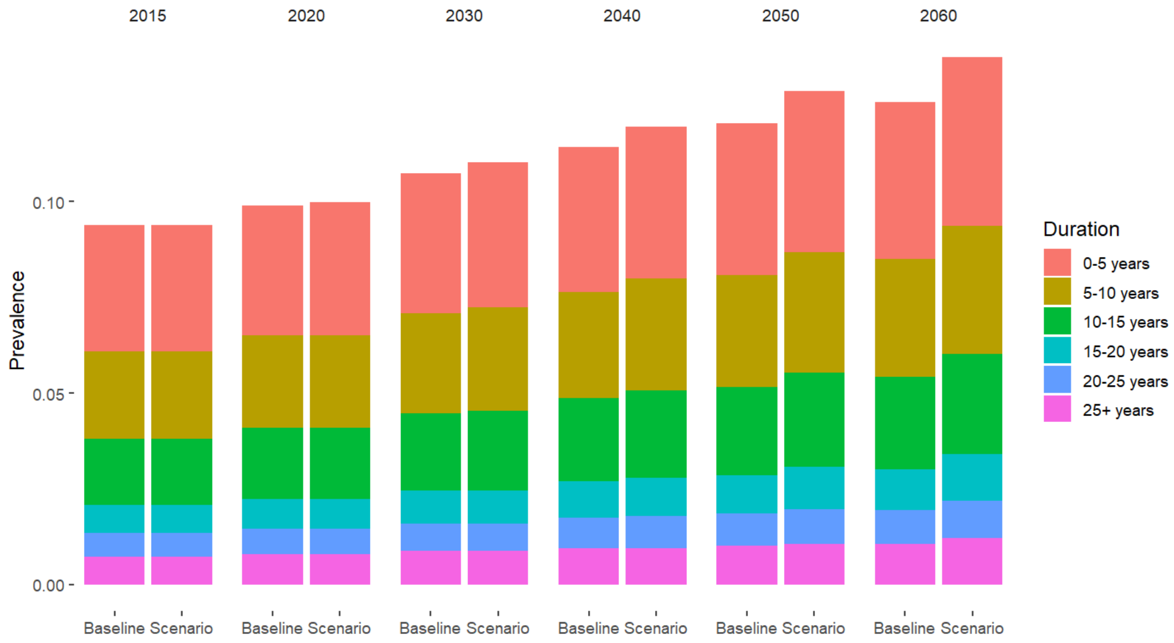

ESM Fig. 20. Prevalence of diabetes of specific duration for baseline scenario and scenario assuming 5% increase in obesity per 5 years. Example for men aged 35-100. Combined prevalence for age 35-100 is calculated as the weighted mean of single-age prevalence using projected population counts as weights.

References:

[1] Guzman-Castillo M, Ahmadi-Abhari S, Bandosz P, et al. (2017) Forecasted trends in disability and life expectancy in England and Wales up to 2025: a modelling study. Lancet Public Health 2(7): e307-e313. 10.1016/S2468-2667(17)30091-9

[2] Ahmadi-Abhari S, Guzman-Castillo M, Bandosz P, et al. (2017) Temporal trend in dementia incidence since 2002 and projections for prevalence in England and Wales to 2040: modelling study. BMJ 358: j2856. 10.1136/bmj.j2856

[3] Currie I, Durban M, Eilers P (2006) Generalized linear array models with applications to multidimensional smoothing. Journal of the Royal Statistical Society Series B-Statistical Methodology 68: 259-280. 10.1111/j.1467-9868.2006.00543.x

[4] Currie I, Durban M, Eilers P (2004) Smoothing and forecasting mortality rates. Statistical Modelling 4(4): 279-298. 10.1191/1471082X04st080oa

[5] Eilers P, Marx B (1996) Flexible smoothing with B-splines and penalties. Statistical Science 11(2): 89-102. 10.1214/ss/1038425655

[6] ONS (2017). 2016-based national population projections consultation papers - Office for National Statistics. Available from https://www.ons.gov.uk/peoplepopulationandcommunity/populationandmigration/populationprojections/methodologies/2016basednationalpopulationprojectionsconsultationpapers. Accessed 19-10-2018

[7] Eddy D, Hollingworth W, Caro J, et al. (2012) Model Transparency and Validation: A Report of the ISPOR-SMDM Modeling Good Research Practices Task Force-7. Medical Decision Making 32(5): 733-743. 10.1177/0272989X12454579

[8] NHS Digital (2015) Health Survey for England, 2015: Trend tables. Available from: https://digital.nhs.uk/data-and-information/publications/statistical/health-survey-for-england/health-survey-for-england-2011-trend-tables. Accessed 20 Nov 2017

[9] WHO (2004) Comparative quantification of health risks. In: WHO Health statistics and information systems. Available from www.who.int/healthinfo/global_burden_disease/cra/en/. Accessed 16 Oct 2018

[10] Brayne C, Gill C, Huppert FA, et al. (1998) Vascular risks and incident dementia: results from a cohort study of the very old. Dementia and geriatric cognitive disorders 9(3): 175-180. 10.1159/000017043

[11] Hassing LB, Johansson B, Nilsson SE, et al. (2002) Diabetes mellitus is a risk factor for vascular dementia, but not for Alzheimer's disease: a population-based study of the oldest old. International psychogeriatrics 14(3): 239-248

[12] Xu W, Caracciolo B, Wang HX, et al. (2010) Accelerated progression from mild cognitive impairment to dementia in people with diabetes. Diabetes 59(11): 2928-2935. 10.2337/db10-0539

[13] Ronnemaa E, Zethelius B, Lannfelt L, Kilander L (2011) Vascular risk factors and dementia: 40-year follow-up of a population-based cohort. Dementia and geriatric cognitive disorders 31(6): 460-466. 10.1159/000330020

[14] Xu W, Qiu C, Gatz M, Pedersen NL, Johansson B, Fratiglioni L (2009) Mid- and late-life diabetes in relation to the risk of dementia: a population-based twin study. Diabetes 58(1): 71-77. 10.2337/db08-0586

[15] Katon W, Pedersen HS, Ribe AR, et al. (2015) Effect of depression and diabetes mellitus on the risk for dementia: a national population-based cohort study. JAMA psychiatry 72(6): 612-619. 10.1001/jamapsychiatry.2015.0082

[16] van den Berg E, de Craen AJ, Biessels GJ, Gussekloo J, Westendorp RG (2006) The impact of diabetes mellitus on cognitive decline in the oldest of the old: a prospective population-based study. Diabetologia 49(9): 2015-2023. 10.1007/s00125-006-0333-1

[17] Ott A, Stolk RP, van Harskamp F, Pols HA, Hofman A, Breteler MM (1999) Diabetes mellitus and the risk of dementia: The Rotterdam Study. Neurology 53(9): 1937-1942

[18] Becker JT, Chang YF, Lopez OL, et al. (2009) Depressed mood is not a risk factor for incident dementia in a community-based cohort. The American journal of geriatric psychiatry : official journal of the American Association for Geriatric Psychiatry 17(8): 653-663
[19] Ganguli M, Fu B, Snitz BE, Hughes TF, Chang CC (2013) Mild cognitive impairment: incidence and vascular risk factors in a population-based cohort. Neurology 80(23): 2112-2120. 10.1212/WNL.0b013e318295d776
[20] Arvanitakis Z, Wilson RS, Bennett DA (2006) Diabetes mellitus, dementia, and cognitive function in older persons. The journal of nutrition, health & aging 10(4): 287-291
[21] Cheng D, Noble J, Tang MX, Schupf N, Mayeux R, Luchsinger JA (2011) Type 2 diabetes and late-onset Alzheimer's disease. Dementia and geriatric cognitive disorders 31(6): 424-430. 10.1159/000324134
[22] Muller M, Tang MX, Schupf N, Manly JJ, Mayeux R, Luchsinger JA (2007) Metabolic syndrome and dementia risk in a multiethnic elderly cohort. Dementia and geriatric cognitive disorders 24(3): 185-192. 10.1159/000105927
[23] Mayeda ER, Haan MN, Kanaya AM, Yaffe K, Neuhaus J (2013) Type 2 diabetes and 10-year risk of dementia and cognitive impairment among older Mexican Americans. Diabetes care 36(9): 2600-2606. 10.2337/dc12-2158
[24] Hayden KM, Zandi PP, Lyketsos CG, et al. (2006) Vascular risk factors for incident Alzheimer disease and vascular dementia: the Cache County study. Alzheimer disease and associated disorders 20(2): 93-100. 10.1097/01.wad.0000213814.43047.86
[25] Akomolafe A, Beiser A, Meigs JB, et al. (2006) Diabetes mellitus and risk of developing Alzheimer disease: results from the Framingham Study. Archives of neurology 63(11): 1551-1555. 10.1001/archneur.63.11.1551
[26] Wu Q, Tchetgen Tchetgen EJ, Osypuk T, et al. (2015) Estimating the cognitive effects of prevalent diabetes, recent onset diabetes, and the duration of diabetes among older adults. Dementia and geriatric cognitive disorders 39(3-4): 239-249. 10.1159/000368654
[27] Alonso A, Mosley TH, Jr., Gottesman RF, Catellier D, Sharrett AR, Coresh J (2009) Risk of dementia hospitalisation associated with cardiovascular risk factors in midlife and older age: the Atherosclerosis Risk in Communities (ARIC) study. Journal of neurology, neurosurgery, and psychiatry 80(11): 1194-1201. 10.1136/jnnp.2009.176818
[28] Exalto LG, Biessels GJ, Karter AJ, Huang ES, Quesenberry CP, Jr., Whitmer RA (2014) Severe diabetic retinal disease and dementia risk in type 2 diabetes. Journal of Alzheimer's disease : JAD 42 Suppl 3: S109-117. 10.3233/jad-132570
[29] Exalto LG, Quesenberry CP, Barnes D, Kivipelto M, Biessels GJ, Whitmer RA (2014) Midlife risk score for the prediction of dementia four decades later. Alzheimer's & dementia : the journal of the Alzheimer's Association 10(5): 562-570. 10.1016/j.jalz.2013.05.1772
[30] Haroon NN, Austin PC, Shah BR, Wu J, Gill SS, Booth GL (2015) Risk of dementia in seniors with newly diagnosed diabetes: a population-based study. Diabetes care 38(10): 1868-1875. 10.2337/dc15-0491
[31] MacKnight C, Rockwood K, Awalt E, McDowell I (2002) Diabetes mellitus and the risk of dementia, Alzheimer's disease and vascular cognitive impairment in the Canadian Study of Health and Aging. Dementia and geriatric cognitive disorders 14(2): 77-83. 10.1159/000064928
[32] Spiers NA, Matthews RJ, Jagger C, et al. (2005) Diseases and impairments as risk factors for onset of disability in the older population in England and Wales: findings from the Medical Research Council Cognitive Function and Ageing Study. The journals of gerontology Series A, Biological sciences and medical sciences 60(2): 248-254
[33] d'Orsi E, Xavier AJ, Steptoe A, et al. (2014) Socioeconomic and lifestyle factors related to instrumental activity of daily living dynamics: results from the English Longitudinal Study of Ageing. Journal of the American Geriatrics Society 62(9): 1630-1639. 10.1111/jgs.12990

[34] Kamper AM, Stott DJ, Hyland M, Murray HM, Ford I (2005) Predictors of functional decline in elderly people with vascular risk factors or disease. Age and ageing 34(5): 450-455. 10.1093/ageing/afi137
[35] Maggi S, Noale M, Gallina P, et al. (2004) Physical disability among older Italians with diabetes. The ILSA study. Diabetologia 47(11): 1957-1962. 10.1007/s00125-004-1555-8
[36] Volpato S, Ferrucci L, Blaum C, et al. (2003) Progression of lower-extremity disability in older women with diabetes: the Women's Health and Aging Study. Diabetes care 26(1): 70-75
[37] Covinsky KE, Hilton J, Lindquist K, Dudley RA (2006) Development and validation of an index to predict activity of daily living dependence in community-dwelling elders. Medical care 44(2): 149-157
[38] Dunlop DD, Semanik P, Song J, Manheim LM, Shih V, Chang RW (2005) Risk factors for functional decline in older adults with arthritis. Arthritis and rheumatism 52(4): 1274-1282. 10.1002/art.20968
[39] Stineman MG, Zhang G, Kurichi JE, et al. (2013) Prognosis for functional deterioration and functional improvement in late life among community-dwelling persons. PM & R : the journal of injury, function, and rehabilitation 5(5): 360-371. 10.1016/j.pmrj.2013.02.008
[40] Wong E, Stevenson C, Backholer K, Woodward M, Shaw JE, Peeters A (2015) Predicting the risk of physical disability in old age using modifiable mid-life risk factors. Journal of epidemiology and community health 69(1): 70-76. 10.1136/jech-2014-204456
[41] Dhamoon MS, Moon YP, Paik MC, Sacco RL, Elkind MS (2014) Diabetes predicts long-term disability in an elderly urban cohort: the Northern Manhattan Study. Annals of epidemiology 24(5): 362-368.e361. 10.1016/j.annepidem.2013.12.013
[42] Gregg EW, Mangione CM, Cauley JA, et al. (2002) Diabetes and incidence of functional disability in older women. Diabetes care 25(1): 61-67
[43] Tooth L, Hockey R, Byles J, Dobson A (2008) Weighted multimorbidity indexes predicted mortality, health service use, and health-related quality of life in older women. Journal of clinical epidemiology 61(2): 151-159. 10.1016/j.jclinepi.2007.05.015
[44] Emerging Risk Factors C, Sarwar N, Gao P, et al. (2010) Diabetes mellitus, fasting blood glucose concentration, and risk of vascular disease: a collaborative meta-analysis of 102 prospective studies. Lancet (London, England) 375(9733): 2215-2222. 10.1016/S0140-6736(10)60484-9
[45] Rao Kondapally Seshasai S, Kaptoge S, Thompson A, et al. (2011) Diabetes mellitus, fasting glucose, and risk of cause-specific death. The New England journal of medicine 364(9): 829-841. 10.1056/NEJMoa1008862
[46] Sarwar N, Gao P, Seshasai SR, et al. (2010) Diabetes mellitus, fasting blood glucose concentration, and risk of vascular disease: a collaborative meta-analysis of 102 prospective studies. Lancet (London, England) 375(9733): 2215-2222. 10.1016/s0140-6736(10)60484-9
[47] Zoungas S, Woodward M, Li Q, et al. (2014) Impact of age, age at diagnosis and duration of diabetes on the risk of macrovascular and microvascular complications and death in type 2 diabetes. Diabetologia 57(12): 2465-2474. 10.1007/s00125-014-3369-7
[48] GOV.UK (2015) Diabetes prevalence estimates for local populations. Available from: https://www.gov.uk/government/publications/diabetes-prevalence-estimates-for-local-populations. Accessed 02 Nov 2017
[49] Government of Canada (2011). Diabetes in Canada: Facts and figures from a public health perspective - Canada.ca. Available from https://www.canada.ca/en/public-health/services/chronic-diseases/reports-publications/diabetes/diabetes-canada-facts-figures-a-public-health-perspective/chapter-1.html, Accessed 05 Nov 2017
